# Supplementary material for: Diagnostic and prognostic performance of the LiverRisk score in tertiary care
Source: JHEP Rep. 2024 Jul 23;6(11):101169. doi: 10.1016/j.jhepr.2024.101169 (PMC11497454; doi:10.1016/j.jhepr.2024.101169)
Supplement: Multimedia component 1 [file mmc1.pdf]

Diagnostic and prognostic performance of the LiverRisk score in tertiary care

Georg Semmler, Lorenz Balcar, Benedikt Simbrunner, Lukas Hartl, Mathias Jachs, Michael Schwarz, Benedikt Silvester Hofer, Laurenz Fritz, Anna Schedlbauer, Katharina Stopfer, Daniela Neumayer, Jurij Maurer, Sophie Gensluckner, Bernhard Scheiner, Elmar Aigner, Michael Trauner, Thomas Reiberger, Mattias Mandorfer

Table of contents

Supplementary results……………………………………………………………….………….2
Fig. S1……………………………………………………………………………………….3
Fig. S2……………………………………………………………………………………….6
Fig. S3……………………………………………………………………………………….8
Fig. S4……………………………………………………………………………………….9
Table S1…………………………………………………………………………………….10
Table S2…………………………………………………………………………………….11
Table S3…………………………………………………………………………………….12
Table S4…………………………………………………………………………………….13

Supplementary results

Regarding patients that were excluded from analysis due to missing laboratory values, the following missingness patterns existed: Of 246 patients excluded from cohort I, 105 (42.7%) were excluded due to missing glucose, 103 (41.9%) due to missing glucose and cholesterol, and 38 (15.4%) due to missing cholesterol, respectively.

Of 81 patients excluded from cohort II, 16 (19.8%) had no laboratory values, 52 (64.2%) had missing ALT/AST, 9 (11.1%) missing platelet count, 2 (2.5%) had missing glucose/cholesterol, one (1.1%) had missing glucose, and another one (1.1%) missing GGT. To compare disease severity, we compared median LSM between included and excluded patients in both cohorts. In cohort I, the median LSM was 6.9 [IQR: 5.1-10.9]kPa for included and 5.9 [IQR: 4.5-7.9]kPa for excluded patients showing a minor but statistically significant difference in terms of a lower LSM in patients with missing laboratory values (Mann-Whitney U test p<0.001). In cohort II, no difference in median LSM was observed (5.8 [IQR: 4.5-8.8]kPa in included vs. 6.5 [IQR: 5.0-10.3]kPa in excluded patients [p=0.090]).

Supplementary figures

Fig. S1. Scatterplot and LOESS curves of LiverRisk score and LSM, FIB-4, and APRI in cohort I (**A**) and cohort II (**B**).

A

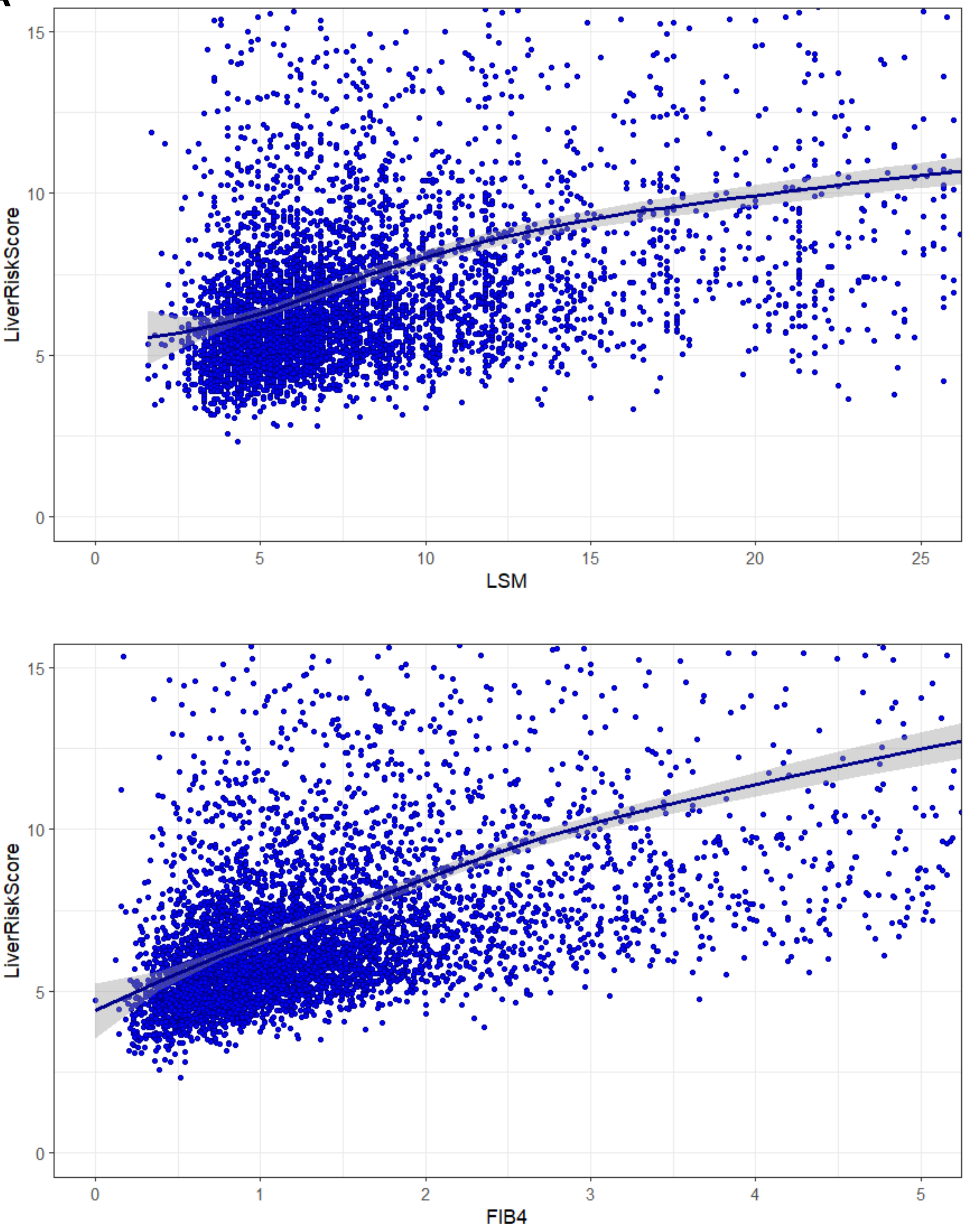

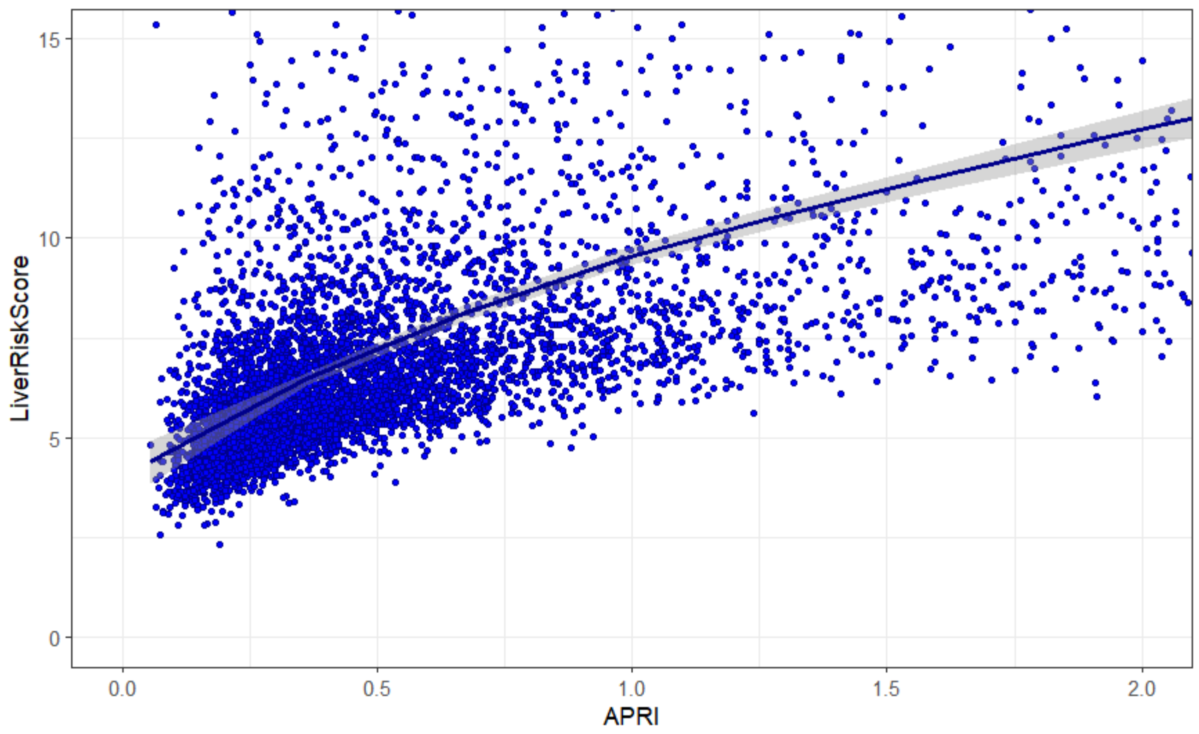

LiverRiskScore
APRI
0
5
10
15
0.0
0.5
1.0
1.5
2.0

B

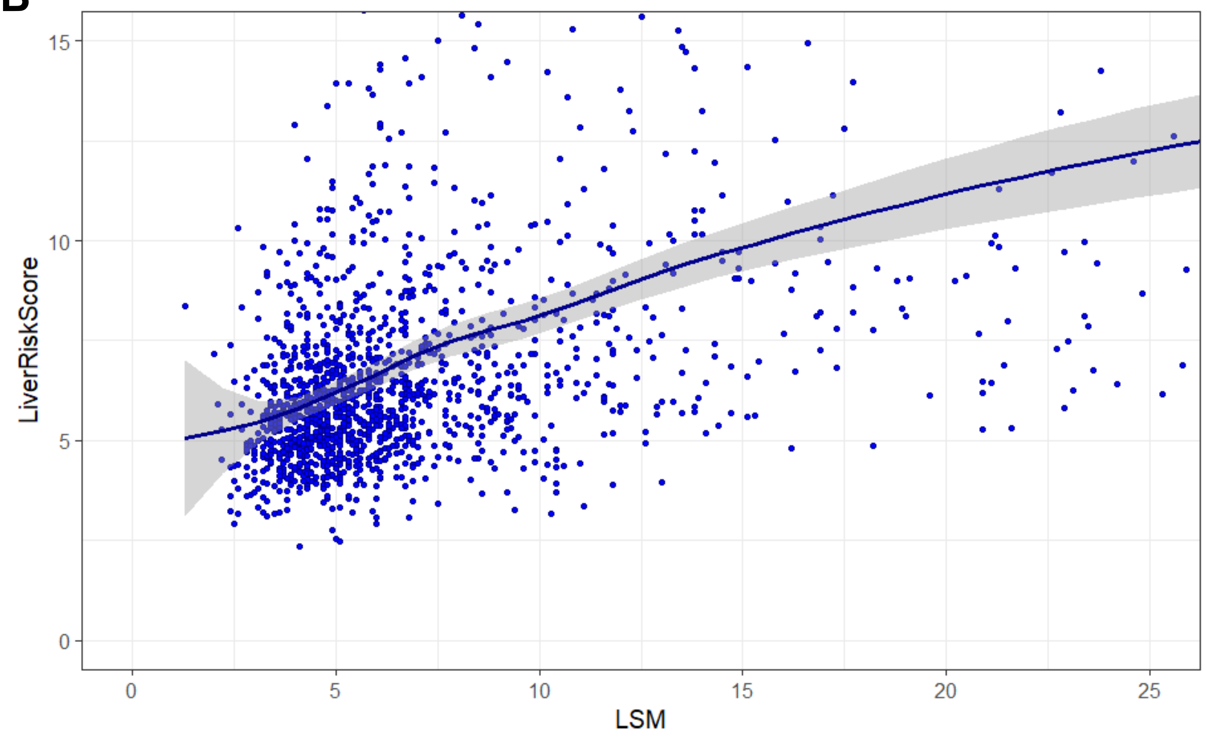

LiverRiskScore
LSM
0
5
10
15
0
5
10
15
20
25

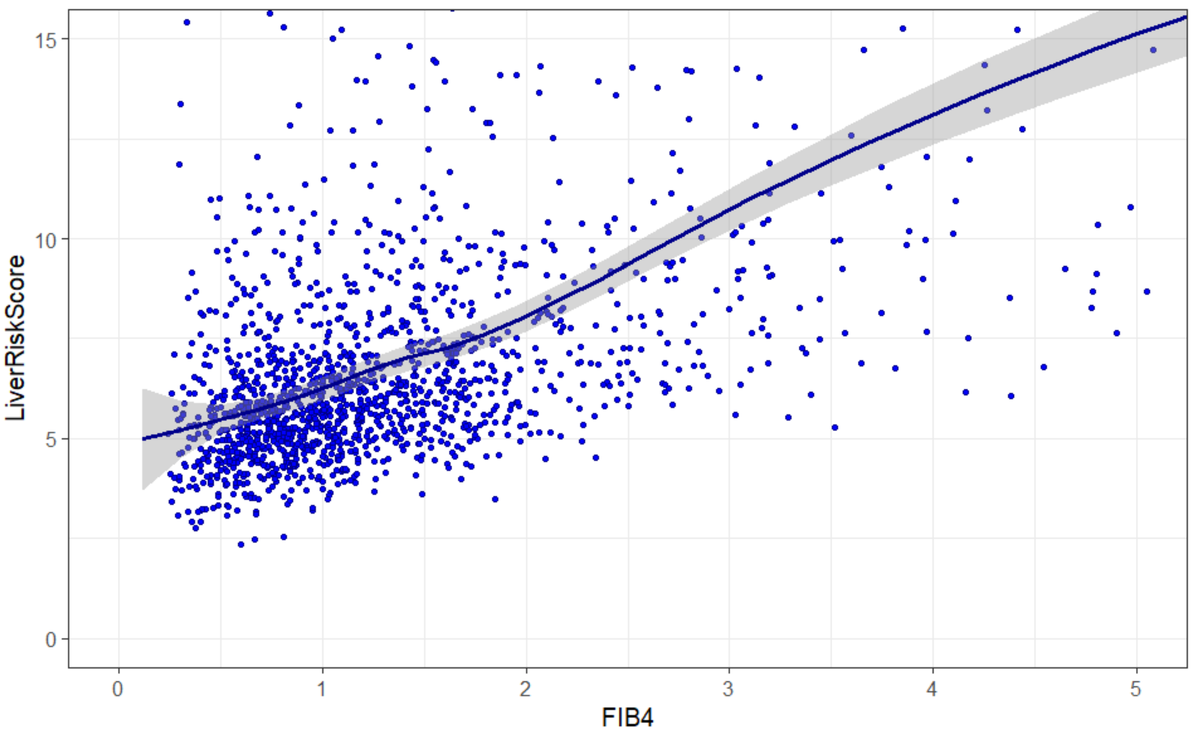

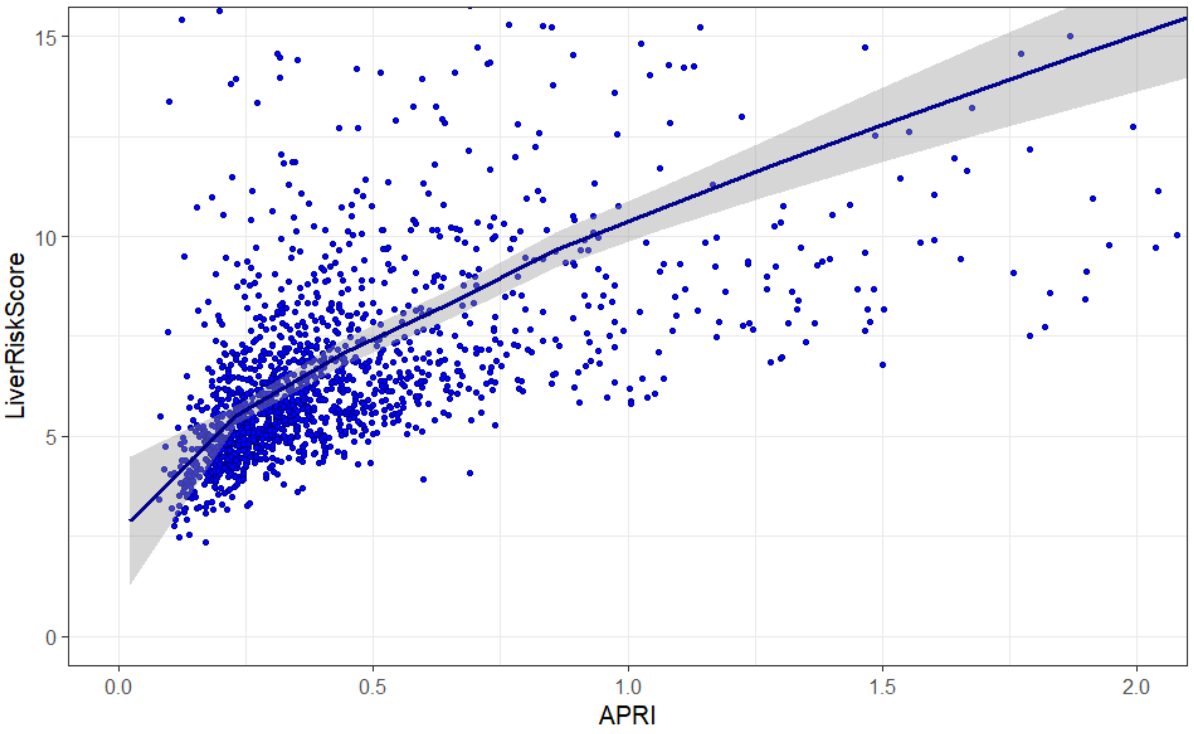

Abbreviations: APRI – aspartate aminotransferase to platelet ratio index; cACLD – compensated advanced chronic liver disease; FIB-4 – fibrosis 4 score; LOESS - locally estimated scatterplot smoothing; LSM – liver stiffness measurement;

Fig. S2. Receiver operating characteristics (ROC) curves for the detection of cACLD (LSM ≥10kPa) using LiverRisk score, FIB-4, and APRI in cohort I (**A**) and cohort II (**B**).

A

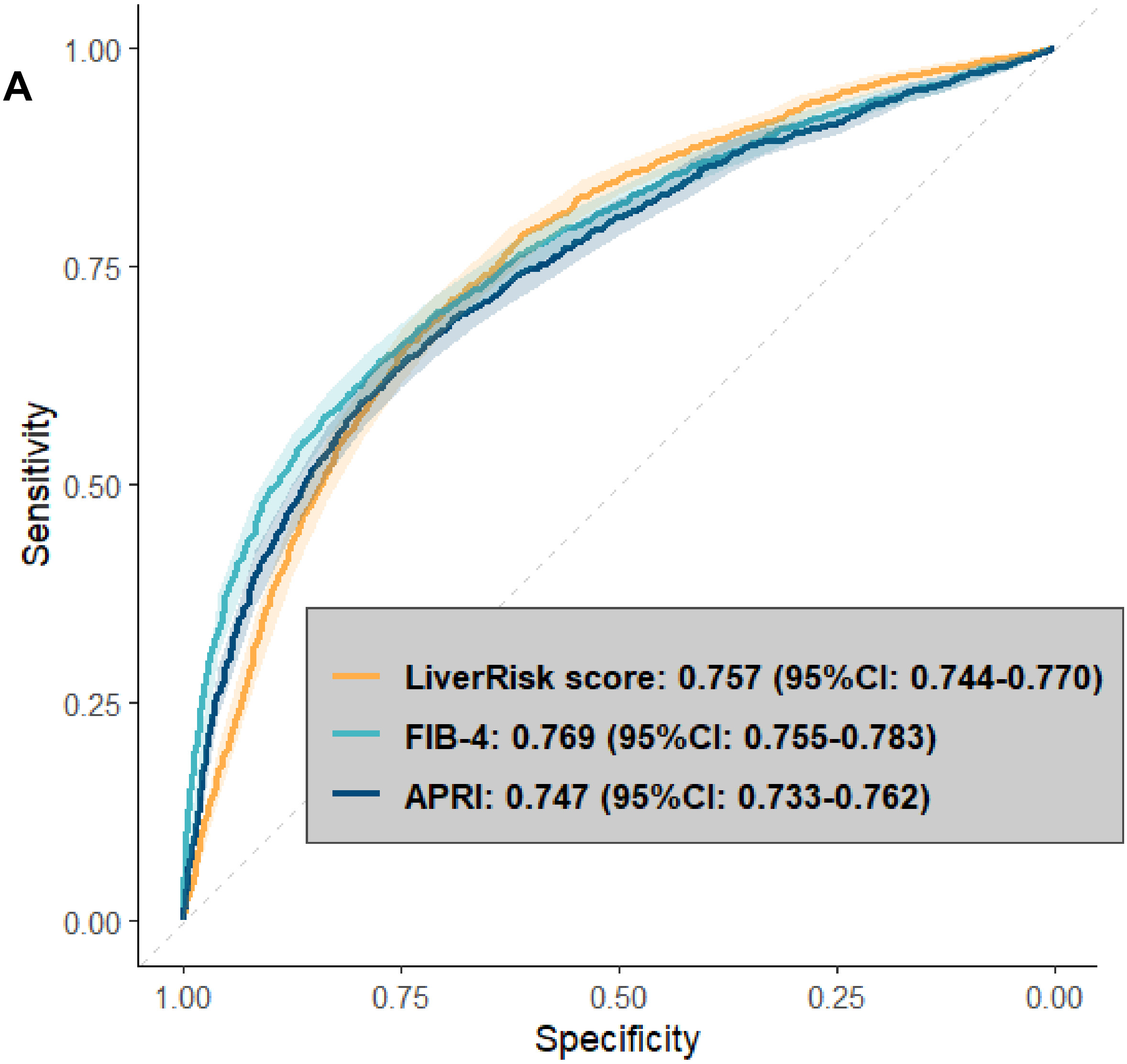

B

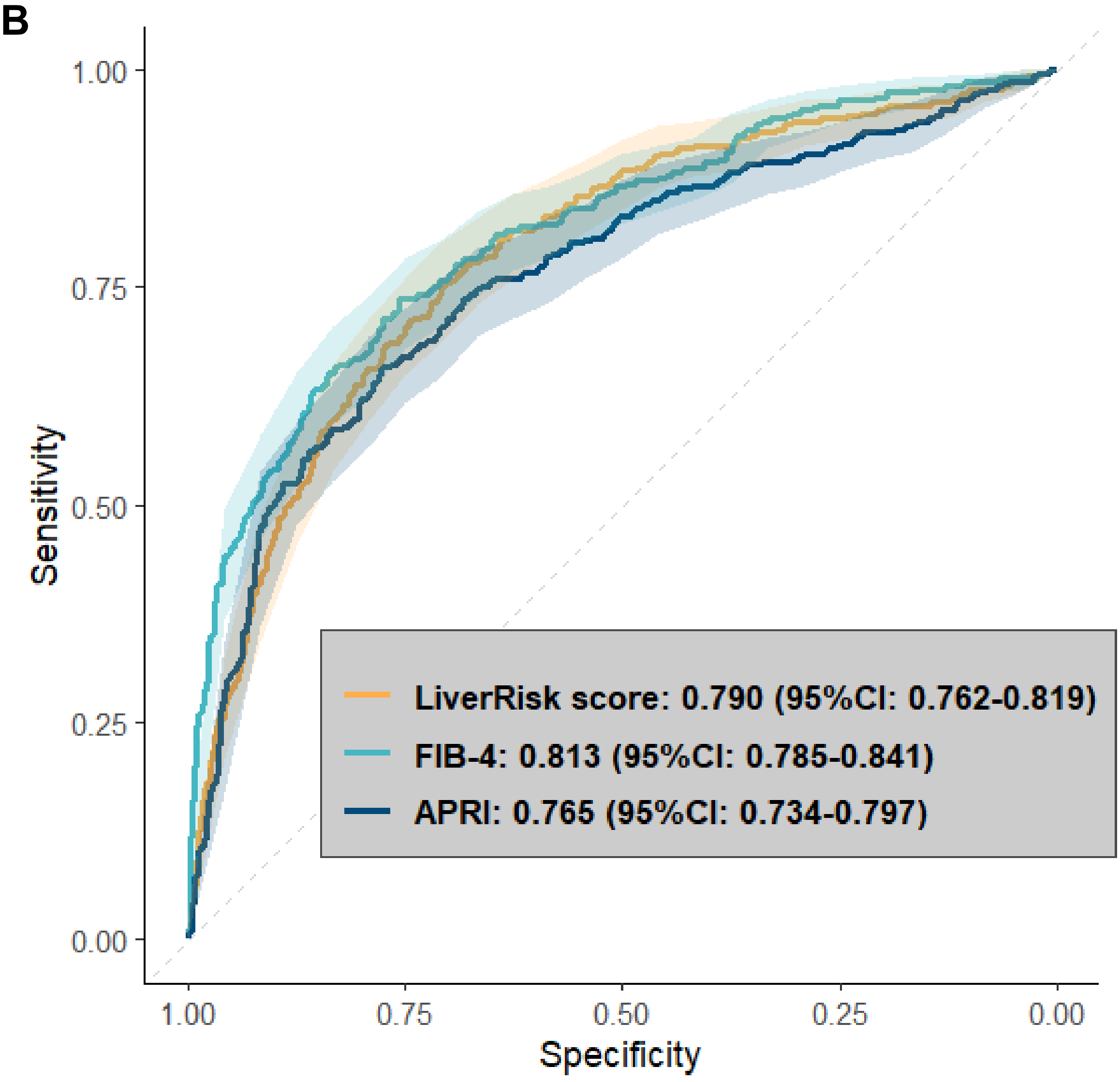

Abbreviations: APRI – aspartate aminotransferase to platelet ratio index; cACLD – compensated advanced chronic liver disease; FIB-4 – fibrosis 4 score; LSM – liver stiffness measurement; ROC – receiver operating characteristics;

Fig. S3. Time-dependent area under the receiver operator characteristics curves (AUROC) and 95% confidence intervals (95%CI) of LiverRisk score, LSM, FIB-4, and APRI for the prediction of hepatic decompensation in cohort I at 1-5 years of follow-up.

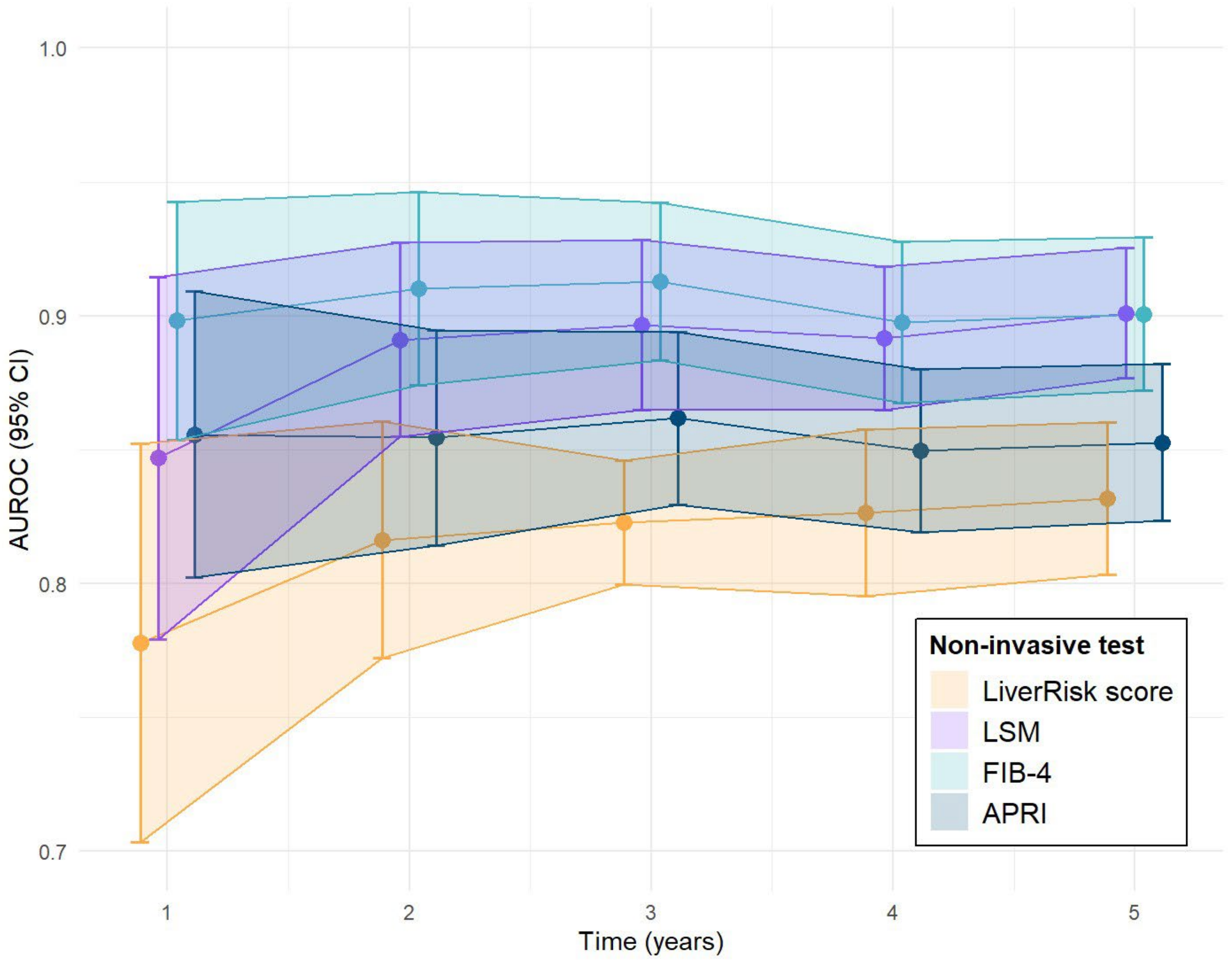

Abbreviations: AUROC – area under the receiver operator characteristics curve; APRI – aspartate aminotransferase to platelet ratio index; cACLD – compensated advanced chronic liver disease; FIB-4 – fibrosis 4 score; LSM – liver stiffness measurement; 95%CI – 95% confidence interval;

Fig. S4. Cumulative incidence curves of hepatic decompensation compared across categories of LiverRisk score (<6, 6-<10, 10-<15, ≥15) in cohort I. Subdistribution hazard ratios (SHR) are given as compared to the first group. (Fine-Gray subdistribution hazard model)

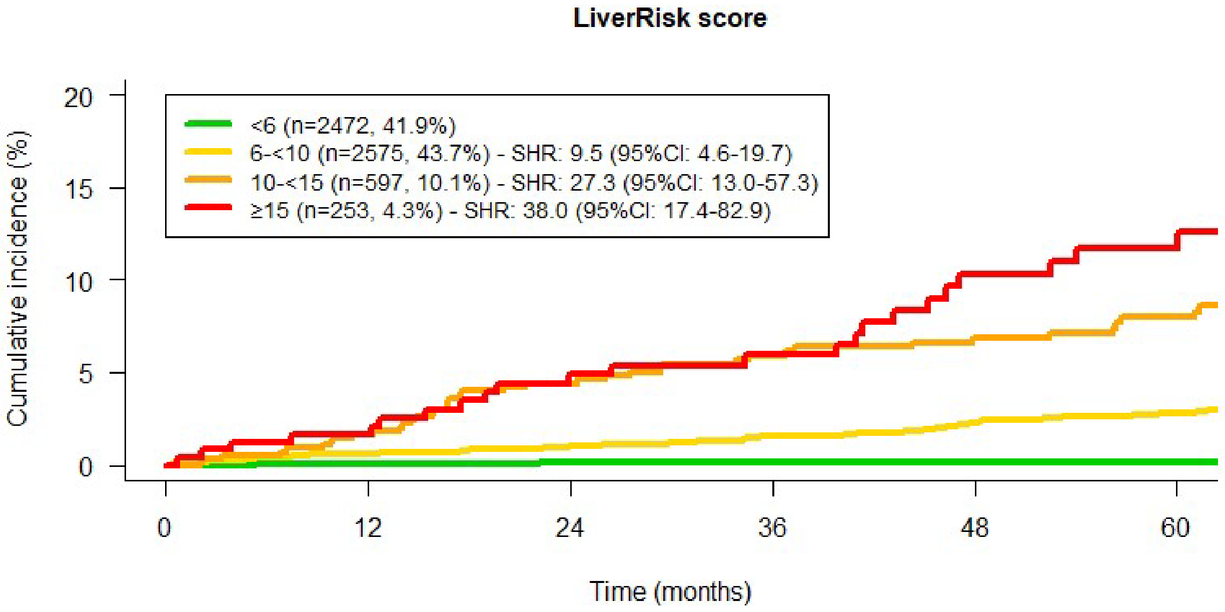

Abbreviations: LSM – liver stiffness measurement; SHR – subdistribution hazard ratio;

Supplementary tables

Table S1. Correlation matrix (Spearman's rank correlation coefficient ρ) for LiverRisk score, LSM, FIB-4, and APRI in cohort I and cohort II.

**Cohort I**	**LiverRisk score**	**LSM**	**FIB-4**	**APRI**
LiverRisk score	1.000	0.469	0.577	0.711
LSM	0.469	1.000	0.448	0.452
FIB-4	0.577	0.448	1.000	0.722
APRI	0.711	0.452	0.722	1.000
**Cohort II**	**LiverRisk score**	**LSM**	**FIB-4**	**APRI**
LiverRisk score	1.000	0.471	0.535	0.684
LSM	0.471	1.000	0.419	0.415
FIB-4	0.535	0.419	1.000	0.617
APRI	0.684	0.415	0.617	1.000

Abbreviations: APRI – aspartate aminotransferase to platelet ratio index; FIB-4 – fibrosis 4 score; LSM – liver stiffness measurement;

Table S2. Cohen's Kappa and 95% confidence intervals (95%CI) between LSM ≥10kPa and LiverRisk score ≥10points in cohort I and cohort II both in the overall cohorts as well as across etiology subgroups.

	**Cohort I, n=5897**	**Cohort II, n=1558**
Overall cohort	0.260 (0.233-0.286)	0.327 (0.268-0.385)
MASLD	0.237 (0.183-0.292)	0.338 (0.257-0.420)
ALD	0.210 (0.110-0.309)	0.337 (0.190-0.483)
Viral	0.255 (0.220-0.290)	0.143 (0.023-0.263)
AIH/Cholestatic	0.309 (0.217-0.401)	0.330 (0.122-0.538)
Other	0.150 (0.030-0.270)	-0.071 (-0.345-0.204)

Abbreviations: AIH – autoimmune hepatitis; ALD – alcohol-related liver disease; LSM – liver stiffness measurement; MASLD – metabolic dysfunction-associated steatotic liver disease; 95%CI – 95% confidence interval;

Table S3. Correlation, calibration, and agreement metrics between the LiverRisk score and LSM, as well as metrics on the diagnostic accuracy of LiverRisk score for the diagnosis of cACLD in cohort I and cohort II in the subgroup of patients meeting reliability criteria for liver fibrosis assessment (≤7kPa or IQR/median <0.3). (Pearson's r, linear regression, Bland-Altmann analysis, ROC analysis)

Out-come	Metric	Cohort I, n=5509	Cohort II, n=1430
LSM (kPa, linear)	Pearson's r (correlation, strong calibration)	0.323	0.431
	R^2 (variation, strong calibration)	0.104	0.185
	Intercept (weak calibration)	4.225 (SE: 0.265)	1.354 (SE: 0.451)
	Slope (weak calibration)	0.775 (SE: 0.031)	0.977 (SE: 0.054)
	Mean difference (95%CI)[1] ≙ Calibration in the large (mean calibration)	2.54 (2.28-2.80)	1.19 (0.75-1.63)
	Lower limit of agreement (95%CI)[1]	-16.87 (-17.32-[-16.42])	-15.57 (-16.33-[-14.82])
	Upper limit of agreement (95%CI)[1]	21.95 (21.50-22.40)	17.96 (17.20-18.71)
	Interval of agreement (kPa/points; 95%CI)[1]	38.82	33.53
cACLD (≥10kPa)	Prevalence (%)	1423 (25.8%)	242 (16.9%)
	AUROC (95%CI)	0.765 (0.751-0.779)	0.816 (0.786-0.845)
	Sensitivity[2] (95%CI)	31.3% (28.9-33.8%)	38.0% (31.9-44.5%)
	Specificity[2] (95%CI)	92.0% (91.1-92.8%)	93.2% (91.6-94.6%)
	Positive predictive value[2] (95%CI)	57.6% (54.4-60.7%)	53.2% (46.6-59.7%)
	Negative predictive value[2] (95%CI)	79.4% (78.8-80.0%)	88.1% (87.0-89.1%)
	Accuracy[2] (95%CI)	76.3% (75.2-77.4%)	83.9% (81.8-85.7%)

[1] based on Bland-Altmann analysis (LSM – LiverRisk score); [2] applying at cut-off of 10points corresponding to 10kPa

Abbreviations: AUROC – area under the receiver operator characteristics curve; cACLD – compensated advanced chronic liver disease; LSM – liver stiffness measurement; ROC – receiver operator characteristics; SE – standard error; 95%CI – 95% confidence interval;

Table S4. Area under the receiver operator characteristics curve (AUROC) and 95% confidence intervals (95%CI) of LiverRisk score, FIB-4 and APRI for the diagnosis of cACLD in cohort I and cohort II, as well as time-dependent AUROC of LiverRisk score, LSM, FIB-4 and APRI for the prediction of hepatic decompensation in cohort I in the subgroup of patients meeting reliability criteria for liver fibrosis assessment (≤7kPa or IQR/median <0.3). (ROC analysis, time-dependent ROC analysis, comparison according to Blanche et al [2013], Level of significance after multiplicity correction: p<0.05)

Outcome	Metric	Cohort I, n=5509	Cohort II, n=1430
cACLD (≥10kPa)	LiverRisk score	0.765 (0.751-0.779)	0.816 (0.786-0.845)
	FIB-4	0.782 (0.767-0.797)	0.826 (0.796-0.857)
	APRI	0.760 (0.745-0.775)	0.781 (0.746-0.816)

Outcome	Metric	Cohort I, n=5509				
Hepatic decompensation	Time	1 year	2 years	3 years	4 years	5 years
	Number of events	24	51	70	92	101
	LiverRisk score	0.772 (0.690-0.855)	0.809 (0.760-0.858)	0.820 (0.782-0.858)	0.829 (0.797-0.862)	0.833 (0.802-0.863)
	LSM	0.860 (0.786-0.934)*	0.898 (0.859-0.937)*	0.904 (0.872-0.937)*	0.901 (0.875-0.928)*	0.906 (0.882-0.931)*
	FIB-4	0.905 (0.860-0.951)*	0.909 (0.869-0.949)*	0.913 (0.881-0.945)*	0.896 (0.864-0.928)*	0.903 (0.874-0.932)*
	APRI	0.862 (0.804-0.919)	0.855 (0.810-0.900)	0.864 (0.829-0.899)	0.851 (0.819-0.882)	0.852 (0.820-0.884)

[*] Indicates a statistically significant difference in AUROC as compared to the LiverRisk score (according to Blanche et al [2013])

Abbreviations: AUROC – area under the receiver operator characteristics curve; cACLD – compensated advanced chronic liver disease; LSM – liver stiffness measurement; ROC – receiver operator characteristics; 95%CI – 95% confidence interval;
