# Supplementary material for: Diagnostic and prognostic performance of the LiverRisk score in tertiary care
Source: JHEP Rep. 2024 Jul 23;6(11):101169. doi: 10.1016/j.jhepr.2024.101169 (PMC11497454; doi:10.1016/j.jhepr.2024.101169)
Supplement: Multimedia component 2 [file mmc2.docx]

**JHEP Reports**

**CTAT methods**

Tables for a “Complete, Transparent, Accurate and Timely account” (CTAT) are now mandatory for all revised submissions. The aim is to enhance the reproducibility of methods.

- 1. **Software**

| **Software name** | **Manufacturer** | **Version** |
| --- | --- | --- |
| R | R Core Team, R Foundation for Statistical Computing | 4.3.2 |

- 1. **Please provide the details of the corresponding methods author for the manuscript:**

| Mattias Mandorfer, MD, PhD |
| --- |
