# Supplementary material for: Diagnostic and prognostic performance of the LiverRisk score in tertiary care
Source: JHEP Rep. 2024 Jul 23;6(11):101169. doi: 10.1016/j.jhepr.2024.101169 (PMC11497454; doi:10.1016/j.jhepr.2024.101169)
Supplement: Multimedia component 4 [file mmc4.pdf]

Diagnostic and prognostic performance of the LiverRisk score in tertiary care☆

Georg Semmler[1,2], Lorenz Balcar[1,2], Benedikt Simbrunner[1,2], Lukas Hartl[1,2], Mathias Jachs[1,2], Michael Schwarz[1,2], Benedikt Silvester Hofer[1,2], Laurenz Fritz[1], Anna Schedlbauer[1], Katharina Stopfer[1], Daniela Neumayer[1], Jurij Maurer[1], Sophie Gensluckner[3], Bernhard Scheiner[1,2], Elmar Aigner[3], Michael Trauner[1], Thomas Reiberger[1,2], Mattias Mandorfer[1,2,*]

JHEP Reports **2024**. vol. 6 | 1–8

Check for updates

Background & Aims: The LiverRisk score has been proposed as a blood-based tool to estimate liver stiffness measurement (LSM), thereby stratifying the risk of compensated advanced chronic liver disease (cACLD, LSM ≥10 kPa) and liver-related events in patients without known chronic liver disease (CLD). We aimed to evaluate its diagnostic/prognostic performance in tertiary care.

Methods: Patients referred to two hepatology outpatient clinics (cohort I, n = 5,897; cohort II, n = 1,558) were retrospectively included. Calibration/agreement of the LiverRisk score with LSM was assessed, and diagnostic accuracy for cACLD was compared with that of fibrosis-4 (FIB-4)/aspartate aminotransferase-to-platelet ratio index (APRI). The prediction of hepatic decompensation and utility of proposed cut-offs were evaluated.

Results: In cohort I/II, mean age was 48.3/51.8 years, 44.2%/44.7% were female, predominant etiologies were viral hepatitis (51.8%)/metabolic dysfunction-associated steatotic liver disease (63.7%), median LSM was 6.9 (IQR 5.1–10.9)/5.8 (IQR 4.5–8.8) kPa, and 1,690 (28.7%)/322 (20.7%) patients had cACLD.
Despite a moderate correlation (Pearson's r = 0.325/0.422), the LiverRisk score systematically underestimated LSM (2.93/1.80 points/kPa lower), and range of agreement was wide, especially at higher values.
The diagnostic accuracy of the LiverRisk score for cACLD (area under the receiver operator characteristics curve [AUROC] 0.757/0.790) was comparable to that of FIB-4 (AUROC 0.769/0.813) and APRI (AUROC 0.747/0.765). The proposed cut-off of 10 points yielded an accuracy of 74.2%/81.2%, high specificity (91.9%/93.4%), but low negative predictive value (76.6%/84.5%, Cohen's κ = 0.260/0.327).
In cohort I, 208 (3.5%) patients developed hepatic decompensation (median follow-up 4.7 years). The LiverRisk score showed a reasonable accuracy for predicting hepatic decompensation within 1–5 years (AUROC 0.778–0.832). However, it was inferior to LSM (AUROC 0.847–0.901, p <0.001) and FIB-4 (AUROC 0.898–0.913, p <0.001). Similar to the strata of other non-invasive tests, the proposed LiverRisk groups had distinct risks of hepatic decompensation.

Conclusions: The LiverRisk score did not improve the diagnosis of cACLD or prediction of hepatic decompensation in the tertiary care setting.

© 2024 The Author(s). Published by Elsevier B.V. on behalf of European Association for the Study of the Liver (EASL). This is an open access article under the CC BY license (http://creativecommons.org/licenses/by/4.0/).

Introduction

Liver disease is the second leading cause of working years of life lost in Europe.[1] Although this calls for earlier interventions, patients often present at late stages after developing complications,[2,3] as the diagnosis of compensated advanced chronic liver disease (cACLD, *i.e.* a spectrum of advanced fibrosis/cirrhosis that confers an increased risk of liver-related events) remains challenging.

Depending on chronic liver disease (CLD) etiology, referral pathways based on staged testing to detect advanced liver fibrosis have been proposed.[4,5] Specifically for metabolic dysfunction-associated steatotic liver disease (MASLD), the most common etiology of CLD, the fibrosis-4 (FIB-4) score has been validated as a sensitive first-line test that is followed by either liver stiffness measurement (LSM) by vibration-controlled transient elastography (VCTE)[6] or patented blood tests for liver fibrosis such as the enhanced liver fibrosis test[7] in case of increased FIB-4 values. However, data on the diagnostic/prognostic performance of non-invasive tests (NITs) in patients without known liver disease remained scarce.

To optimize screening in the general population, the LiverScreen Consortium developed the LiverRisk score to predict LSM and thus stratify the risk of cACLD and associated complications.[8] This score was developed in a pooled cohort of

☆ Given their role as Editor, Mattias Mandorfer had no involvement in the peer-review of this article and had no access to information regarding its peer-review. Full responsibility for the editorial process for this article was delegated to the Guest Editor Ian Rowe.

* Corresponding author. Address: Division of Gastroenterology and Hepatology, Department of Medicine III, Medical University of Vienna, Währinger Gürtel 18-20, 1090, Vienna, Austria. Tel.: +43-1-40400-47440; Fax: +43-1-40400-47350.
E-mail address: mattias.mandorfer@meduniwien.ac.at (M. Mandorfer).

https://doi.org/10.1016/j.jhepr.2024.101169

6,357 patients with LSM from seven prospective cohorts and showed superior discriminatory ability for cACLD as compared with FIB-4 or aspartate aminotransferase-to-platelet ratio index (APRI). When stratifying patients from the UK Biobank into four risk groups (<6, 6 to <10, 10 to <15, and ≥15 points), liver-related events occurred primarily in the high- and medium-risk groups. Although the LiverRisk score was intended for opportunistic screening of liver fibrosis among patients seen in primary care with metabolic risk factors for CLD or chronic alcohol consumption, the authors discuss automatically including it in lab reports from hospitals and health centers, raising the question of whether the LiverRisk score may be applicable in settings other than those where it was derived. Here, the LiverRisk score could serve as an alternative risk stratification tool to VCTE, as it is based on simple and readily available blood tests and its assessment does not require specific expertise/trained personnel or elastography resources, which may be increasingly overwhelmed by referrals for steatotic liver disease (SLD). Thus, we evaluated the diagnostic and prognostic utility of the LiverRisk score in patients referred to two hepatology outpatient clinics.

Patients and methods

Patient cohorts

In cohort I, 5,897 patients with known/suspected CLD (*i.e.* referred for diagnostic workup and/or clinical care) undergoing laboratory assessment and LSM at the Medical University Vienna from 2007 to 2020 were retrospectively included at their first LSM. Patients were excluded if they had a history of hepatic decompensation, hepatocellular carcinoma (HCC), orthotopic liver transplantation, vascular liver disease (*i.e.* causes of prehepatic, presinusoidal, or posthepatic portal hypertension), cystic fibrosis-associated liver disease, congestive hepatopathy, congenital metabolic diseases, sarcoidosis, secondary sclerosing cholangitis, impaired liver function (*i.e.* Child–Turcotte–Pugh stage B/C), no clinical follow-up, or insufficient clinical data. Finally, patients were excluded if they had missing laboratory values to calculate the LiverRisk score (n = 246, 4.0%; *i.e.* complete case analysis). Demographic and clinical characteristics were assessed at the time of first LSM, whereas laboratory data were obtained within 3 months (all at the same day). Data on hepatic decompensation until December 2022 were obtained from medical records using manual chart review. Data on survival were complemented by a systematic query of the national death registry.

In cohort II, 1,558 patients who attended the hepatology outpatient clinic of the Paracelsus Medical University Salzburg for the first time between June 2016 and July 2020 were included at first referral. The exclusion criteria were identical to those for cohort I. In total, n = 81 (4.9%) patients were excluded because of missing laboratory values to calculate the LiverRisk score. Demographic characteristics and clinical and laboratory data were assessed at the day of LSM.

Further details on excluded patients are presented in the Supplementary information.

Objectives

The primary objective was to study the diagnostic accuracy of the LiverRisk score for cACLD (LSM ≥10 kPa) in comparison with other blood-based NITs. Secondary objectives included calibration for LSM, agreement with LSM, and the prognostic accuracy for predicting hepatic decompensation.

Liver stiffness measurement

LSM by VCTE (FibroScan®, Echosens, Paris, France) was performed by experienced operators adhering to established quality criteria.[9] All LSMs were performed under fasting conditions. Applying published reliability criteria for the assessment of liver fibrosis (IQR/median <0.3 or ≤7.0 kPa), 5,509 (93.4%) LSMs in cohort I and 1,430 (91.8%) in cohort II met these criteria. For all patients, the first LSM after referral to the tertiary care center was used. An LSM ≥10 kPa denoted cACLD. For prognostic purposes, patients were stratified according to LSM cut-offs <10, 10–14.9, and ≥15 kPa.

Blood-based NITs

The FIB-4 and APRI scores were calculated as previously described.[10,11] For group comparison, patients were stratified according to broadly used FIB-4 (<1.3, 1.3–2.67, and >2.67) and APRI (<0.5, 0.5–1.5, and >1.5 points) cut-offs. The LiverRisk score was calculated using the provided online calculator (https://www.liverriskscore.com/multicalc) combining age, sex, fasting glucose, cholesterol, aspartate aminotransferase (AST), alanine transaminase (ALT), gamma-glutamyltransferase (GGT), and platelet count (PLT).[8] Risk groups included minimal (<6), low (6 to <10), medium (10 to <15), and high (≥15) risks corresponding to 10 and 15 kPa to exclude or rule-in cACLD.

Outcomes

cACLD was defined as LSM ≥10 kPa.[12] Hepatic decompensation was defined as the first occurrence of clinically apparent ascites, variceal bleeding, or overt hepatic encephalopathy.[12] HCC was diagnosed according to the respective clinical practice guidelines of the EASL.[13]

Ethics

The study was approved by the local ethics committee of the Medical University of Vienna (1029/2023) and Paracelsus Medical University Salzburg (1026/2021), and it was performed in conformity with the current version of the Helsinki Declaration. The requirement of written informed consent was waived by the institutional review board.

Statistical analyses

Statistical analyses were performed using R 4.3.2 (R Core Team, R Foundation for Statistical Computing, Vienna, Austria). Continuous variables were reported as mean ± SD or median and IQR, whereas categorical variables were reported as proportion of patients with/without a certain characteristic.

Because the LiverRisk score was designed to estimate LSM (kPa) by VCTE (FibroScan®), linear regression analysis was used to study their relationship and evaluate calibration of LiverRisk $\sim$ LSM.[14] First, mean calibration was assessed as the mean difference between LSM and the LiverRisk score (see also Bland–Altman analysis). Calibration slope and intercept were derived from the linear regression model. ‘Moderate’ calibration was investigated graphically by using natural splines

with three degrees of freedom and inspecting the deviation from the linear regression line. Finally, R^2 (*i.e.* variation/proportion of variation in LSM explained by the LiverRisk score) and Pearson's correlation coefficient r (*i.e.* standardized regression coefficient β) were provided.

Bland–Altman analyses were performed to assess the agreement between LSM and the LiverRisk score, including the mean difference (*i.e.* bias ≙ calibration in the large) and the lower and upper limits of agreement (*i.e.* 95% confidence interval [CI] of agreement corresponding to ±1.96 × SD of observed difference).

Spearman's rank correlation coefficient (ρ) was used to study the correlation between the LiverRisk score and other lab-based NITs (FIB-4 and APRI), applying locally estimated scatterplot smoothing (LOESS) to graphically display non-linear relationships. The area under the receiver operator characteristics curve (AUROC) of different NITs for cACLD (*i.e.* LSM ≥10 kPa) was calculated and compared using the DeLong test (pROC-package). Cohen's kappa was used to quantify agreement between LSM ≥10 kPa and LiverRisk score ≥10 points categories. Time-dependent AUROCs for the prediction of hepatic decompensation during the first 5 years of follow-up were computed using the timeROC-package, adjusting for competing risks and applying inverse probability of censoring weighting, and compared according to Blanche *et al.*[15] using multiplicity correction as provided by the timeROC-package. Hepatic decompensation after diagnosis of HCC was not considered. Median follow-up was calculated using the reverse Kaplan–Meier method.[16]

Cumulative incidence functions were used to study the incidence of hepatic decompensation during follow-up while accounting for HCC and death as competing risks (cmprsk-package). Cumulative incidences were compared using Fine–Gray competing risks regression models. For these analyses, non-invasive scores were categorized as previously proposed. A p value <0.05 was considered statistically significant.

Table 1. Patient characteristics of cohorts I and II.

Patient characteristics	Cohort I (n = 5,897)	Cohort II (n = 1,558)
Age (years)	48.3 ± 14.2	51.8 ± 15.5
Female sex	2,604 (44.2%)	697 (44.7%)
BMI* (kg/m^2)	25.9 (22.8–30.1)	26.0 (23.2–29.4)
Diabetes	839 (14.2%)	157 (10.1%)
Etiology		
AIH/cholestatic	482 (8.2%)	114 (7.3%)
ALD	350 (5.9%)	145 (9.3%)
MASLD	1,688 (28.6%)	984 (63.2%)
Viral	3,053 (51.8%)	280 (18.0%)
Other	324 (5.5%)	35 (2.2%)
LSM (kPa)	6.9 (5.1–10.9)	5.8 (4.5–8.8)
<6	2,187 (37.1%)	803 (51.5%)
6–9.9	2,020 (34.3%)	433 (27.8%)
10–14.9	781 (13.2%)	149 (9.6%)
≥15	909 (15.4%)	173 (11.1%)
LSM ≥10 kPa (cACLD)	1,690 (28.7%)	322 (20.7%)
LiverRisk score (points)	6.38 (5.30–8.23)	6.17 (5.13–7.95)
<6	2,472 (41.9%)	716 (46.0%)
6 to <10	2,575 (43.7%)	649 (41.7%)
10 to <15	597 (10.1%)	129 (8.3%)
≥15	253 (4.3%)	64 (4.1%)
Platelet count (G/L)	221 (174–268)	240 (200–283)
AST (U/L)	35 (25–54)	33 (26–47)
ALT (U/L)	43 (27–73.0)	40 (26–65)
GGT (U/L)	52 (25–118)	59 (28–129)
Glucose (mg/dl)	94 (86–106)	93 (85–104)
Cholesterol (mg/dl)	177 (151–207)	199 (169–232)
APRI	0.41 (0.26–0.73)	0.35 (0.25–0.56)
<0.5	3,533 (59.9%)	1,093 (70.2%)
0.5–1.5	1,792 (30.4%)	392 (25.2%)
>1.5	572 (9.7%)	73 (4.7%)
FIB-4	1.19 (0.77–1.90)	1.11 (0.75–1.70)
<1.3	3,276 (55.6%)	925 (59.4%)
1.3–2.67	1,741 (29.5%)	453 (29.1%)
>2.67	880 (14.9%)	180 (11.6%)

AIH, autoimmune hepatitis; ALD, alcohol-related liver disease; ALT, alanine aminotransferase; APRI, aspartate aminotransferase-to-platelet ratio index; AST, aspartate aminotransferase; FIB-4, fibrosis-4; GGT, gamma-glutamyl transferase; LSM, liver stiffness measurement; MASLD, metabolic dysfunction-associated steatotic liver disease.
*Missing in 603 (10.2%) and 37 (2.4%) patients in cohorts I and II, respectively.

Results

Patient characteristics

In cohort I, 5,897 patients (mean age 48.3 ± 14.2 years, 2,604 [44.2%] female) were included (Table 1). Predominant etiologies were viral hepatitis (n = 3,053, 51.8%) and MASLD (n = 1,688, 28.6%). Median LSM was 6.9 (IQR 5.1–10.9) kPa, corresponding to 2,187 (37.1%) patients with LSM <6 kPa, 2,020 (34.3%) with LSM 6–9.9 kPa, and 1,690 (28.7%) with LSM ≥10 kPa (*i.e.* suggestive of cACLD), of whom 909 (15.4%) had LSM ≥15 kPa. Median LiverRisk score was 6.38 (IQR 5.30–8.23), with 2,472 (41.9%) patients allocated to the minimal-risk group (<6 points), 2,575 (43.7%) to the low-risk group (6 to <10 points), 597 (10.1%) to the medium-risk group (10 to <15 points), and 253 (4.3%) to the high-risk group (≥15 points).

In cohort II, of the 1,558 patients included (mean age 51.8 ± 15.5 years, 697 [44.7%] female), 984 (63.2%) had MASLD, and 280 (18.0%) had viral hepatitis. Median LSM was slightly lower (5.8 [IQR 4.5–8.8] kPa), corresponding to 803 (51.5%) patients with LSM <6 kPa, 433 (27.8%) with LSM 6–9.9 kPa, and 322 (20.7%) with LSM ≥10 kPa, of whom 173 (11.1%) had LSM ≥15 kPa. Median LiverRisk score was 6.17 (IQR 5.13–7.95), with 716 (46.0%) patients allocated to the minimal-risk group (<6 points), 649 (41.7%) to the low-risk group (6 to <10), 129 (8.3%) to the medium-risk group (10 to <15), and 64 (4.1%) to the high-risk group (≥15).

Correlation, calibration, and agreement between LSM and the LiverRisk score

LSM and the LiverRisk score showed a moderate correlation (Pearson's r = 0.325/0.422 in cohort I/II) (Table 2). Although their correlation was rather logarithmic in cohort I, possibly leading to the lower Pearson's r, it was nearly linear in cohort II (Fig. 1). In both cohorts, the LiverRisk score tended to underestimate LSM, which was less pronounced in cohort II (intercept of linear regression ≙ calibration curve 4.502/1.788 points/kPa). Although the calibration slope was 1.002 in cohort II, the predicted LSM values were more extreme in cohort I (slope 0.791).

Next, the agreement between LSM and the LiverRisk score was assessed using the Bland–Altman method and plot (Table 2 and Fig. 2). Again, the mean difference (bias) between LSM and the LiverRisk score (LSM minus LiverRisk score) was 2.927 and 1.802 points/kPa, respectively, indicating overall underestimation (LSM on average was higher than the

Table 2. Correlation, calibration, and agreement metrics between the LiverRisk score and LSM, as well as metrics on the diagnostic accuracy of LiverRisk score for the diagnosis of cACLD in cohorts I and II.

Metric	Cohort I (n = 5,897)	Cohort II (n = 1,558)
**LSM (kPa, linear)**		
Pearson's r	0.325	0.422
R^2	0.106	0.178
Intercept (SE)	4.502 (0.260)	1.788 (0.457)
Slope (SE)	0.791 (0.030)	1.002 (0.055)
Mean difference (95% CI)* ≙ calibration in the large (mean calibration)	2.93 (2.67–3.18)	1.80 (1.36–2.25)
Lower limit of agreement (95% CI)*	-16.75 (-17.19 to [-16.31])	-15.82 (-16.58 to [-15.06])
Upper limit of agreement (95% CI)*	22.61 (22.17–23.04)	19.42 (18.66–20.19)
Interval of agreement (kPa/points)*	39.36	35.24
**cACLD (≥10 kPa)**		
Prevalence, n (%)	1,690 (28.7)	322 (20.7)
AUROC (95% CI)	0.757 (0.743–0.770)	0.790 (0.762–0.819)
Sensitivity† (95% CI) (%)	30.2 (28.0–32.4)	34.5 (29.3–39.9)
Specificity† (95% CI) (%)	91.9 (91.1–92.7)	93.4 (91.8–94.7)
Positive predictive value† (95% CI) (%)	60.7 (57.0–63.0)	57.6 (51.2–63.7)
Negative predictive value† (95% CI) (%)	76.6 (76.0–77.2)	84.5 (83.4–85.5)
Accuracy† (95% CI) (%)	74.2 (73.1–75.3)	81.2 (79.1–83.1)

These include Pearson's r, linear regression, Bland–Altmann analysis, ROC analysis.
AUROC, area under the receiver operator characteristics curve; cACLD, compensated advanced chronic liver disease; LSM, liver stiffness measurement.
*Based on Bland–Altmann analysis (LSM - LiverRisk score).
†Applying at a cut-off of 10 points, corresponding to 10 kPa.

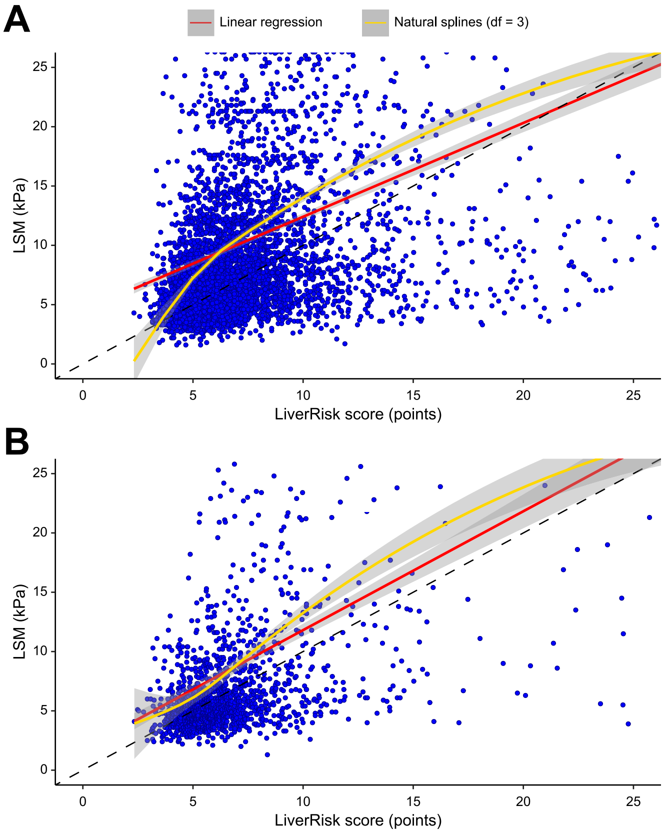

Fig. 1. Scatterplot of the LiverRisk score and LSM in (A) cohort I and (B) cohort II. The dashed black line represents the 'identity line', corresponding to perfect calibration between LSM and the LiverRisk score (*i.e.* predicted LSM); the red line represents the linear fit (see Table 2); and the yellow line represents a non-linear fit with natural splines and three degrees of freedom (df) for (more) flexible modeling of the relationship between LSM and the LiverRisk score. Figures were cut at 25 kPa/points for better interpretability; therefore, further outliers are not displayed. LSM, liver stiffness measurement.

LiverRisk score). Most importantly, the range of differences was considerable (95% CI of agreement 39.36 and 35.24 points/kPa) and showed a pattern toward larger disagreement at higher values, with the LiverRisk score being lower than LSM.

Accuracy of the LiverRisk score for diagnosing cACLD

The LiverRisk score had a moderate diagnostic accuracy for cACLD (LSM ≥10 kPa) in cohort I (AUROC 0.757, 95% CI 0.744–0.770) but was slightly higher in cohort II (AUROC 0.790, 95% CI 0.762–0.819) (Table 2). Applying the proposed cut-off of 10 points corresponding to 10 kPa yielded a high specificity (91.9%/93.4%) in both cohorts yet a negative predictive value of 76.6%/84.5%, indicating that one in four to five patients with a LiverRisk score of <10 points still had cACLD. However, sensitivity was low (30.2%/34.5%). Fig. 3 shows the proportion of concordant results in terms of risk categories (LSM ≥10 kPa and LiverRisk score ≥10 points), corresponding to an overall accuracy of 74.2%/81.2%. Importantly, no clustering regarding disease etiologies was evident. In line with our findings on calibration (*i.e.* underestimation of LSM by the LiverRisk score), false negatives for cACLD were particularly common in cohort I (20.0%) and were still considerably more common than false positives in cohort II (13.5% *vs.* 5.3%). Cohen's kappa, which quantified the agreement between LSM ≥10 kPa and LiverRisk score ≥10 points, indicated a higher agreement in cohort II (κ = 0.327, 95% CI 0.268–0.385) than in cohort I (κ = 0.260, 95% CI 0.233–0.286). Agreement was lower but comparable across alcohol-related liver disease, MASLD, and viral etiologies in cohort I, whereas in cohort II, agreement was particularly low in viral etiologies (Table S2).

Comparison with blood-based NITs (FIB-4 and APRI)

In both cohorts, the LiverRisk score showed a moderate to strong correlation with FIB-4 (ρ = 0.577/0.535) and APRI (ρ = 0.711/0.684) (Table S1). Using local regression, the

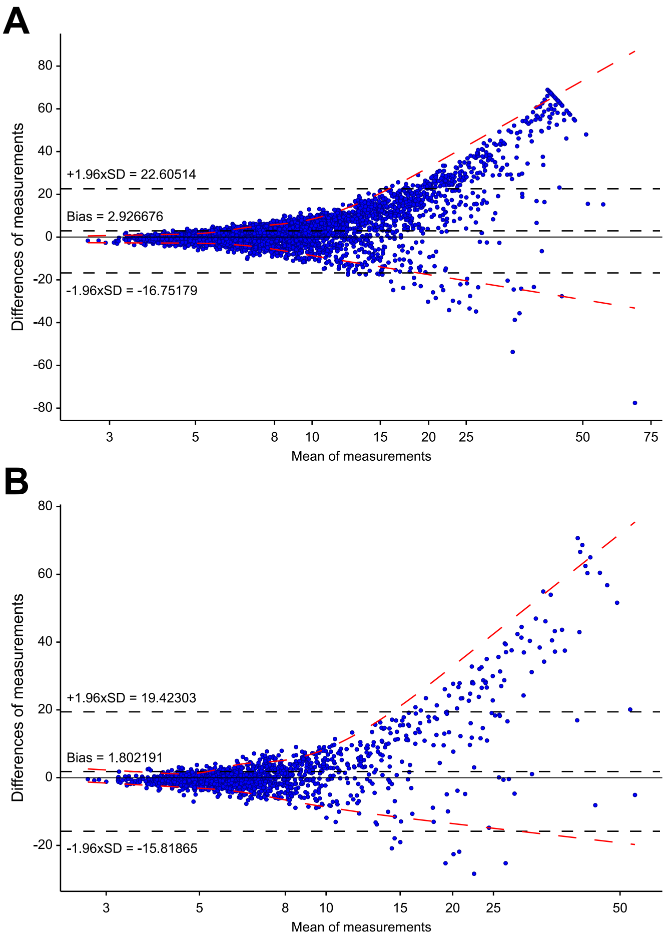

Fig. 2. Modified Bland–Altman plot showing the mean of LSM and LiverRisk score within a patient (x-axis) and the difference (LSM - LiverRisk score, y-axis) in (A) cohort I and (B) cohort II. Dashed black lines represent the mean difference of both measurements (*i.e.* calibration in the large, 'bias') and the upper and lower limits of agreement, corresponding to the area of agreement within ±1.96 times the SD of the difference observed in the respective sample. Values close to 0 indicate no difference within the same subject (*i.e.* agreement). Red dashed lines represent quantile regression of the 95% CI of differences–means, modeled with natural splines (df = 3). The x-axis was log-transformed to increase interpretability in the clinically relevant range 5–15 kPa. df, degrees of freedom; LSM, liver stiffness measurement.

relationships of the LiverRisk score with FIB-4 and APRI were nearly linear (Fig. S1).

Regarding the accuracy to diagnose cACLD, FIB-4 and APRI were comparable to the LiverRisk score in cohort I (AUROC for FIB-4 0.769, 95% CI 0.755–0.783, DeLong's test p = 0.081; AUROC for APRI 0.747, 95% CI 0.733–0.762, p = 0.099) and cohort II (AUROC for FIB-4 0.831, 95% CI 0.785–0.841, p = 0.099; AUROC for APRI 0.765, 95% CI 0.734–0.797, p = 0.064) (Table 3 and Fig. S2).

Prediction of hepatic decompensation

Median follow-up in cohort I was 4.7 (IQR 2.2–7.3) years, during which 208 (3.5%) patients developed hepatic decompensation, 90 (1.5%) developed HCC, and 562 (9.5%) died.

For the prediction of hepatic decompensation within 5 years of follow-up, the LiverRisk score showed a good prognostic accuracy (AUROC 0.778–0.832 from 1 to 5 years) (Table 3 and Fig. S3). However, other NITs (LSM and FIB-4) showed statistically significant better (AUROC for LSM 0.847–0.901; AUROC for FIB-4 0.898–0.913) or comparable performance (AUROC for APRI 0.850–0.862).

Risk stratification using established cut-offs

When applying the proposed cut-offs for the LiverRisk score in cohort I, patients allocated to the minimal-risk group (<6 points; n = 2,472, 41.9%) had a negligible risk of hepatic decompensation (0.2% at 5 years) (Fig. S4). In contrast, patients in the low-risk group (n = 2,575, 43.7%) had a significantly higher risk (subdistribution hazard ratio [SHR] 9.5, 95% CI 4.6–19.7) with 2.8% developing hepatic decompensation at 5 years. Risk increased substantially in the medium-risk group (n = 597, 10.1%; SHR 27.3, 95% CI 13.0–57.3; 8.0% hepatic decompensation at 5 years) and the high-risk group (n = 253, 4.3%; SHR 38.0, 95% CI 17.4–82.9; 11.7% hepatic decompensation at 5 years).

However, when comparing the discrimination with other NITs (LSM, FIB-4, and APRI) by applying similar cut-offs, it was evident that discrimination was comparable, if not inferior, to LSM (<10 kPa, ≥15 kPa), FIB-4 (<1.3, >2.67), and APRI (<0.5, >1.5) in this setting (Fig. 4). Importantly, the LiverRisk score assigned <10 points to a large group of patients (n = 5,047, 85.6%) who still had a considerable risk of hepatic decompensation (1.5% at 5 years), whereas it was 0.2% for FIB-4 <1.3 and 0.3% for LSM <10 kPa or APRI <1.5.

Subgroup analysis in patients meeting reliability criteria for LSM

In the subgroup of patients meeting reliability criteria of LSM for the assessment of liver fibrosis, results were comparable, and these are displayed in Tables S3 and S4.

Discussion

The present study applies the newly developed LiverRisk score in patients with suspected CLD to evaluate its diagnostic/prognostic performance outside the original publication in a different clinically important patient group.[8] Studying two large cohorts (n = 5,897 and 1,558) recruited at major tertiary hepatology outpatient clinics in Austria, we demonstrate suboptimal calibration of the LiverRisk score for LSM, with considerable discordance in this setting and a moderate accuracy in diagnosing cACLD and predicting hepatic decompensation, being comparable to well-established, simpler, and potentially cheaper (*i.e.* less laboratory tests needed) blood-based NITs such as FIB-4 and APRI.

As a first step, we studied calibration and agreement between LSM and the LiverRisk score. Most importantly, results from the Bland–Altman-analyses, which is an established method to assess agreement between two metric variables, showed a considerable variation in the difference between LSM and the LiverRisk score, with 95% of measurements differentiating within an interval of ~40 kPa/points (±20 kPa/points). Considering that decisions on whether to refer a patient to a hepatology clinic and/or pursue further diagnostics usually depend on smaller differences in LSM, this variation should be considered from a clinical point of view and raises concerns regarding its applicability in tertiary settings. Despite data from

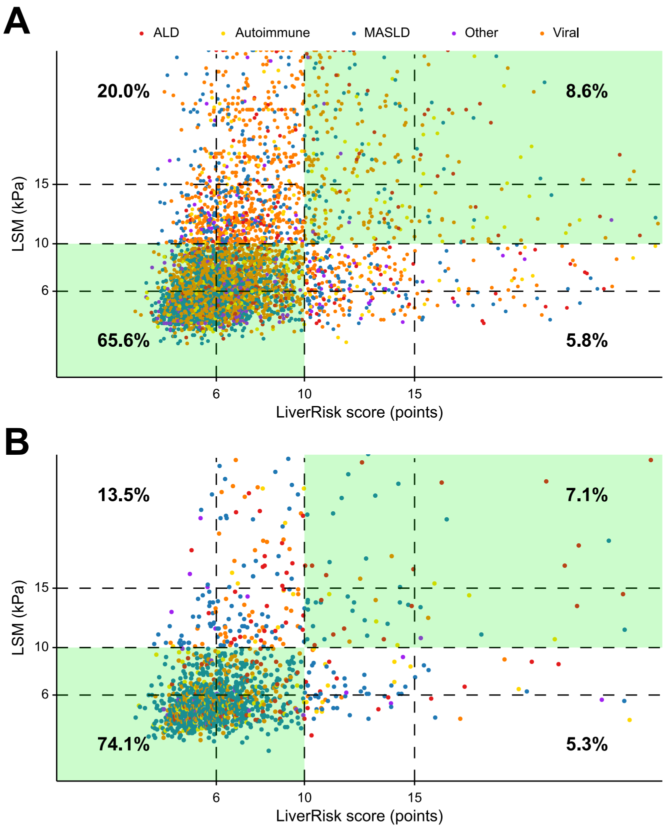

Fig. 3. Scatterplot of the LiverRisk score and LSM in (A) cohort I and (B) cohort II focusing on the distribution of disease etiologies (colored groups) and agreement/concordance for cACLD (cut-off ≥10 kPa/points). ALD, alcohol-related liver disease; cACLD, compensated advanced chronic liver disease; LSM, liver stiffness measurement; MASLD, metabolic dysfunction-associated steatotic liver disease.

cohort II (20.7% prevalence of cACLD and 63.2% MASLD) indicating better overall calibration, the variability was similar and must be considered before clinical application in an individual patient.

Importantly, the LiverRisk score was comparable to FIB-4 and APRI in terms of its diagnostic performance for cACLD and to APRI in predicting hepatic decompensation. However, FIB-4 was superior to the LiverRisk score in the latter context. This is important, as APRI/FIB-4 scores are universally applicable in primary, secondary, and tertiary care, with numerous studies supporting their use. In contrast, the use of the LiverRisk score, despite a moderate to strong correlation with FIB-4/APRI, should currently be limited to the primary care setting. Notably, even in the latter context, its large-scale implementation is currently limited by the unavailability of the formula.

In general, differences in the accuracy of the LiverRisk score outside the primary care setting may be explained by several factors. First, the setting in which we applied the LiverRisk score was different from the setting for which it was developed (tertiary *vs.* primary care). Nevertheless, age, BMI, and the prevalence of diabetes were comparable between our cohorts and the derivation cohort. In addition, median LSM was similar (6.9 kPa/5.8 kPa *vs.* 5.9 kPa in the derivation cohort), although the prevalence of cACLD is expected to be higher (28.7%/20.7% in cohort I/II, not stated for the derivation cohort of the LiverRisk score).[8] Here, different prevalences of the primary outcome need to be acknowledged, as they may influence sensitivity and specificity via spectrum bias.[17] However, some cohorts merged in the LiverRisk score derivation cohort might not fully represent the general population/primary care setting, as they included pre-selected risk groups overlapping with the current study: one study included only patients from hospital liver clinics and alcohol rehabilitation centers,[18] another included only patients with risk factors for CLD (alcohol abuse, diabetes, and elevated ALT),[19] and a third included only patients with known metabolic risk factors.[20] As laboratory fibrosis tests such as FIB-4 have not been and are currently not implemented in primary care in Austria, patients with mildly elevated liver enzymes and/or abnormal ultrasound findings are referred to hepatology clinics without any prefiltering/restrictions, potentially leading to considerable overlaps in the background populations between the derivation cohort and our cohorts. Second, etiologies of liver disease were different between the cohorts. While the derivation cohort included patients 'without known liver disease' (as discussed above), cohort I mainly comprised patients with viral hepatitis (51.8%). However, the more contemporary cohort II consisted

Table 3. AUROCs and 95% CIs of LiverRisk score, FIB-4, and APRI for the diagnosis of cACLD in cohorts I and II, as well as time-dependent AUROC values of LiverRisk score, LSM, FIB-4, and APRI for the prediction of hepatic decompensation in cohort I.

Metric			Cohort I (n = 5,897)		Cohort II (n = 1,558)
**cACLD (≥10 kPa)**					
LiverRisk score			0.757 (0.744–0.770)		0.790 (0.762–0.819)
FIB-4			0.769 (0.755–0.783)		0.831 (0.785–0.841)
APRI			0.747 (0.733–0.762)		0.765 (0.734–0.797)
			**Cohort I (n = 5,897)**		
**Hepatic decompensation**					
Time (years)	1	2	3	4	5
Events (n)	28	60	79	102	115
LiverRisk score	0.778 (0.703–0.852)	0.816 (0.772–0.860)	0.823 (0.787–0.858)	0.826 (0.795–0.858)	0.832 (0.803–0.860)
LSM	0.847 (0.779–0.915)*	0.891 (0.855–0.927)*	0.897 (0.865–0.928)*	0.892 (0.865–0.918)*	0.901 (0.877–0.925)*
FIB-4	0.898 (0.854–0.943)*	0.910 (0.874–0.946)*	0.913 (0.883-0.942)*	0.898 (0.868–0.928)*	0.901 (0.872–0.929)*
APRI	0.856 (0.802–0.909)	0.855 (0.814–0.895)	0.862 (0.829–0.894)	0.850 (0.819–0.880)	0.853 (0.824–0.882)

ROC analysis, time-dependent ROC analysis, and comparison according to Blanche *et al.*[15] Level of significance after multiplicity correction: p <0.05.
AUROC, area under the receiver operator characteristics curve; APRI, aspartate aminotransferase-to-platelet ratio index; cACLD, compensated advanced chronic liver disease; FIB-4, fibrosis-4; LSM, liver stiffness measurement; ROC, receiver operator characteristics.
*Statistically significant difference in AUROC compared with the AUROC of LiverRisk score (according to Blanche *et al.*[15]).

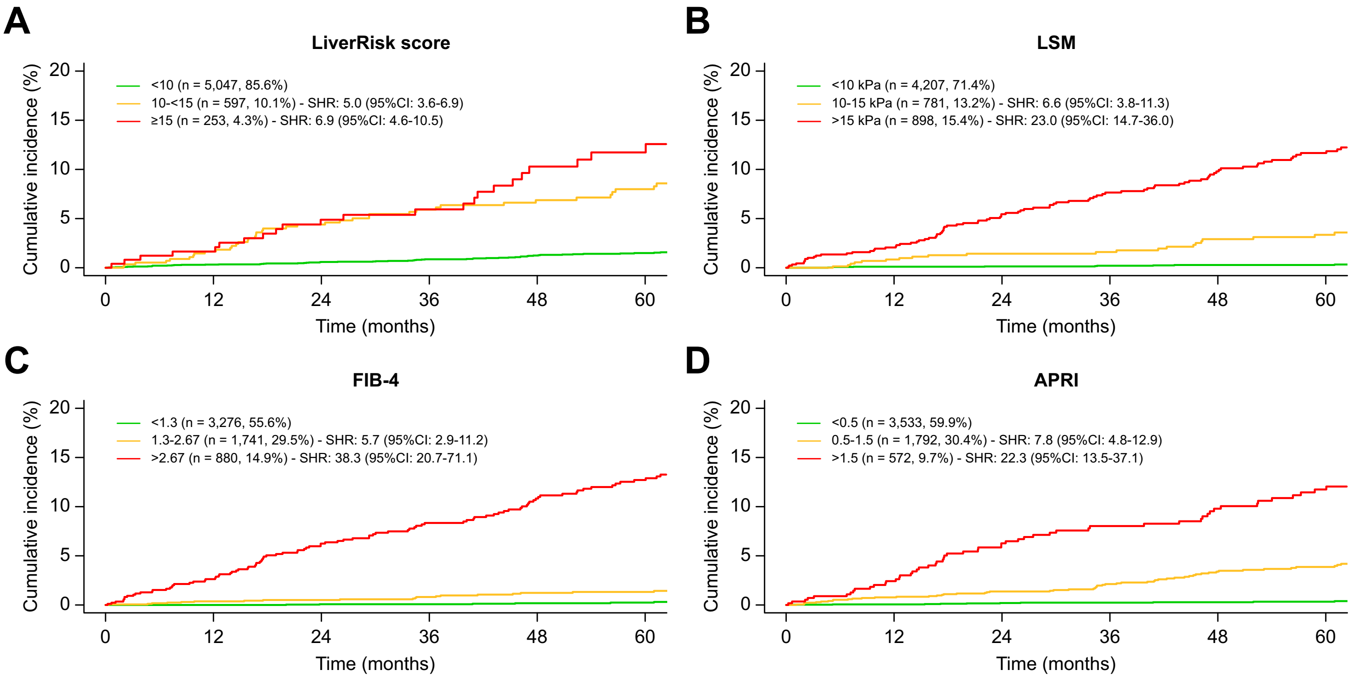

Fig. 4. Cumulative incidence curves of hepatic decompensation compared across categories of (A) LiverRisk score (<10, 10 to <15, and ≥15), (B) LSM (<10, 10–15, and >15 kPa), (C) FIB-4 (<1.3, 1.3–2.67, and >2.67), and (D) APRI (<0.5, 0.5–1.5, and >1.5) in cohort I. SHRs are given compared with the first group (Fine–Gray subdistribution hazard model). APRI, aspartate aminotransferase-to-platelet ratio index; CI, confidence interval; FIB-4, fibrosis-4; LSM, liver stiffness measurement; SHR, subdistribution hazard ratio.

predominantly of patients with MASLD (63.2%) and still showed a comparable diagnostic accuracy of the LiverRisk score and FIB-4/APRI.

Although the authors clearly state that the score is intended for use in the general population, they also discuss its implementation in periodic laboratory controls in patients with chronic conditions in hospitals or health centers.[8] However, as these represent different patient populations, the utility of the LiverRisk score in the latter context still needed to be evaluated. We demonstrate that the LiverRisk score does not improve cACLD identification in tertiary care, with a comparable to inferior predictive performance for hepatic decompensation as compared with FIB-4 and LSM, with the latter having been extensively validated in clinical practice.[21–23] This finding is important, as it indicates that physicians in tertiary care settings with a higher pre-test probability of cACLD can rely on established NITs (*e.g.* LSM or FIB-4), whereas the LiverRisk seems to be less suited.

This study has several limitations. Although laboratory assessment and LSM have been performed systematically at both clinics at first referral, LSM and a complete set of laboratory parameters necessary for calculating the LiverRisk score were not available in all patients because of the retrospective design of our study. Second, because of the high proportion of viral hepatitis in cohort I, it is not fully representative of the contemporary spectrum of patients followed at tertiary care liver clinics. However, this limitation was ameliorated by including cohort II, in which comparable findings were observed. Third, it is difficult to differentiate between patients with or without known liver disease, as there is no general consensus about how to deal with patients referred due to suspicion of liver disease but in whom the diagnosis has yet to be established. Finally, although blood testing is usually performed under fasting conditions, it cannot be guaranteed that glucose levels were drawn under fasting conditions in all our patients. Yet the available data mirror clinical practice/real-life.

In summary, the LiverRisk score showed considerable discrepancies with the observed LSM and may therefore not (yet) be applicable in settings other than primary care. The LiverRisk score was not superior to FIB-4 and APRI in diagnosing cACLD in patients with known or suspected liver disease from tertiary care, and its prognostic relevance was inferior to that of FIB-4 (and LSM). Thus, although it represents a major step forward for screening patients without known liver disease in primary care, our findings indicate that the LiverRisk score does not improve patient management outside the primary care setting, that is, in cohorts with a higher pre-test probability of cACLD.

Affiliations

[1]*Division of Gastroenterology and Hepatology, Department of Medicine III, Medical University of Vienna, Vienna, Austria;* [2]*Vienna Hepatic Hemodynamic Lab, Division of Gastroenterology and Hepatology, Department of Medicine III, Medical University of Vienna, Vienna, Austria;* [3]*Department of Medicine, Medical University of Vienna, Vienna, Austria*

Abbreviations

ALT, alanine transaminase; APRI, aspartate aminotransferase-to-platelet ratio index; AST, aspartate aminotransferase; AUROC, area under the receiver operator characteristics curve; cACLD, compensated advanced chronic liver disease; CI, confidence interval; CLD, chronic liver disease; FIB-4, fibrosis-4; GGT, gamma-glutamyltransferase; HCC, hepatocellular carcinoma; LOESS, locally estimated scatterplot smoothing; LSM, liver stiffness measurement; MASLD, metabolic dysfunction-associated steatotic liver disease; NIT, non-invasive test; PLT, platelet count; ROC, receiver operating characteristic; VCTE, vibration controlled transient elastography.

Financial support

This work was supported by a grant from the 'Ärztekammer für Wien' awarded to GS (0023-WS2020).

Conflicts of interest

LB, LH, BSH, LF, AS, KS, DN, JM, RS, SG, and EA have nothing to disclose. GS received travel support from Amgen. BSi received travel support from AbbVie and Gilead. MJ served as speaker/consultant for Gilead. MS received travel support from MSD, Sandoz, BMS, AbbVie, and Gilead and speaking honoraria from BMS. BS received travel support from AbbVie, Ipsen, and Gilead. MT received grant support from Albireo, Alnylam, Cymabay, Falk, Gilead, Intercept, MSD, Takeda, and UltraGenyx; honoraria for consulting from Abbvie, Albireo, Boehringer Ingelheim, BiomX, Falk, Genfit, Gilead, Hightide, Intercept, Jannsen, MSD, Novartis, Phenex, Pliant, Regulus, Siemens, and Shire; speaker fees from Albireo, Bristol-Myers Squibb, Falk, Gilead, Intercept, MSD, and Madrigal; and travel support from AbbVie, Falk, Gilead, and Intercept. He is also co-inventor on patents on the medical use of norUDCA/norucholic acid filed by the Medical University of Vienna. TR received grant support from AbbVie, Boehringer Ingelheim, Gilead, MSD, Gore, Philips Healthcare, Pliant Pharmaceuticals, and Siemens; speaking honoraria from AbbVie, Gilead, Gore, Intercept, Roche, and MSD; consulting/advisory board fee from AbbVie, Bayer, Boehringer Ingelheim, Gilead, Intercept, MSD, and Siemens; and travel support from Boehringer Ingelheim, Gilead, and Roche. MM served as a speaker and/or consultant and/or advisory board member for AbbVie, Collective Acumen, Echosens, Gilead, Ipsen, Takeda, and W. L. Gore & Associates and received travel support from AbbVie and Gilead.

Please refer to the accompanying ICMJE disclosure forms for further details.

Authors' contributions

Study conception: GS, MM. Data collection: all authors. Statistical analysis: GS, MM. Drafting of the manuscript: GS, MM. Review for important intellectual content and approval of the final manuscript: all authors.

Data availability statement

Data are available from the authors upon reasonable request.

Supplementary data

Supplementary data to this article can be found online at https://doi.org/10.1016/j.jhepr.2024.101169.

References

Author names in bold designate shared co-first authorship

[1] Karlsen TH, Sheron N, Zelber-Sagi S, et al. The EASL–Lancet Liver Commission: protecting the next generation of Europeans against liver disease complications and premature mortality. Lancet 2022;399:61–116.

[2] D'Amico G, Garcia-Tsao G, Pagliaro L. Natural history and prognostic indicators of survival in cirrhosis: a systematic review of 118 studies. J Hepatol 2006;44:217–231.

[3] **Balcar L**, **Tonon M**, Semmler G, et al. Risk of further decompensation/mortality in patients with cirrhosis and ascites as the first single decompensation event. JHEP Rep 2022;4:100513.

[4] European Association for the Study of the Liver. EASL Clinical Practice Guidelines on non-invasive tests for evaluation of liver disease severity and prognosis—2021 update. J Hepatol 2021;75:659–689.

[5] **Semmler G**, **Hartl L**, Mendoza YP, et al. Simple blood tests to diagnose compensated advanced chronic liver disease and stratify the risk of clinically significant portal hypertension. Hepatology 2024. https://doi.org/10.1097/HEP.0000000000000829.

[6] Mózes FE, Lee JA, Selvaraj EA, et al. Diagnostic accuracy of non-invasive tests for advanced fibrosis in patients with NAFLD: an individual patient data meta-analysis. Gut 2022;71:1006–1019.

[7] Kjaergaard M, Lindvig KP, Thorhauge KH, et al. Using the ELF test, FIB-4 and NAFLD fibrosis score to screen the population for liver disease. J Hepatol 2023;79:277–286.

[8] Serra-Burriel M, Juanola A, Serra-Burriel F, et al. Development, validation, and prognostic evaluation of a risk score for long-term liver-related outcomes in the general population: a multicohort study. Lancet 2023;402:988–996.

[9] Schwabl P, Bota S, Salzl P, et al. New reliability criteria for transient elastography increase the number of accurate measurements for screening of cirrhosis and portal hypertension. Liver Int 2015;35:381–390.

[10] Sterling RK, Lissen E, Clumeck N, et al. Development of a simple noninvasive index to predict significant fibrosis in patients with HIV/HCV coinfection. Hepatology 2006;43:1317–1325.

[11] Wai CT, Greenson JK, Fontana RJ, et al. A simple noninvasive index can predict both significant fibrosis and cirrhosis in patients with chronic hepatitis C. Hepatology 2003;38:518–526.

[12] de Franchis R, Bosch J, Garcia-Tsao G, et al. Baveno VII—renewing consensus in portal hypertension. J Hepatol 2022;76:959–974.

[13] European Association for the Study of the Liver. EASL Clinical Practice Guidelines: management of hepatocellular carcinoma. J Hepatol 2018;69:182–236.

[14] Van Calster B, Nieboer D, Vergouwe Y, et al. A calibration hierarchy for risk models was defined: from utopia to empirical data. J Clin Epidemiol 2016;74:167–176.

[15] Blanche P, Dartigues JF, Jacqmin-Gadda H. Estimating and comparing time-dependent areas under receiver operating characteristic curves for censored event times with competing risks. Stat Med 2013;32:5381–5397.

[16] Schemper M, Smith TL. A note on quantifying follow-up in studies of failure time. Control Clin Trials 1996;17:343–346.

[17] Usher-Smith JA, Sharp Stephen J, Griffin SJ. The spectrum effect in tests for risk prediction, screening, and diagnosis. BMJ 2016;353:i3139.

[18] Thiele M, Detlefsen S, Sevelsted Moller L, et al. Transient and 2-dimensional shear-wave elastography provide comparable assessment of alcoholic liver fibrosis and cirrhosis. Gastroenterology 2016;150:123–133.

[19] Harman DJ, Ryder SD, James MW, et al. Direct targeting of risk factors significantly increases the detection of liver cirrhosis in primary care: a cross-sectional diagnostic study utilising transient elastography. BMJ Open 2015;5:e007516.

[20] Fabrellas N, Hernández R, Graupera I, et al. Prevalence of hepatic steatosis as assessed by controlled attenuation parameter (CAP) in subjects with metabolic risk factors in primary care. a population-based study. PLoS One 2018;13:e0200656.

[21] Semmler G, Yang Z, Fritz L, et al. Dynamics in liver stiffness measurements predict outcomes in advanced chronic liver disease. Gastroenterology 2023;165:1041–1052.

[22] Thorhauge KH, Semmler G, Johansen S, et al. Using liver stiffness to predict and monitor the risk of decompensation and mortality in patients with alcohol-related liver disease. J Hepatol 2024;81:23–32.

[23] **Semmler G**, **Alonso López S**, Pons M, et al. Post-treatment LSM rather than change during treatment predicts decompensation in patients with cACLD after HCV cure. J Hepatol 2024;81:76–83.

Keywords: FIB-4; Liver stiffness measurement; LSM; cACLD; Chronic liver disease.

Received 14 January 2024; received in revised form 2 July 2024; accepted 15 July 2024; Available online 23 July 2024

Journal of Hepatology, Volume *6*

Supplemental information

Diagnostic and prognostic performance of the LiverRisk score in tertiary care

Georg Semmler, Lorenz Balcar, Benedikt Simbrunner, Lukas Hartl, Mathias Jachs, Michael Schwarz, Benedikt Silvester Hofer, Laurenz Fritz, Anna Schedlbauer, Katharina Stopfer, Daniela Neumayer, Jurij Maurer, Sophie Gensluckner, Bernhard Scheiner, Elmar Aigner, Michael Trauner, Thomas Reiberger, and Mattias Mandorfer

Diagnostic and prognostic performance of the LiverRisk score in tertiary care

Georg Semmler, Lorenz Balcar, Benedikt Simbrunner, Lukas Hartl, Mathias Jachs, Michael Schwarz, Benedikt Silvester Hofer, Laurenz Fritz, Anna Schedlbauer, Katharina Stopfer, Daniela Neumayer, Jurij Maurer, Sophie Gensluckner, Bernhard Scheiner, Elmar Aigner, Michael Trauner, Thomas Reiberger, Mattias Mandorfer

Table of contents

Supplementary results……………………………………………………………………2
Fig. S1………………………………………………………………………………………3
Fig. S2………………………………………………………………………………………6
Fig. S3………………………………………………………………………………………8
Fig. S4………………………………………………………………………………………9
Table S1……………………………………………………………………………………10
Table S2……………………………………………………………………………………11
Table S3……………………………………………………………………………………12
Table S4……………………………………………………………………………………13

Supplementary results

Regarding patients that were excluded from analysis due to missing laboratory values, the following missingness patterns existed: Of 246 patients excluded from cohort I, 105 (42.7%) were excluded due to missing glucose, 103 (41.9%) due to missing glucose and cholesterol, and 38 (15.4%) due to missing cholesterol, respectively.

Of 81 patients excluded from cohort II, 16 (19.8%) had no laboratory values, 52 (64.2%) had missing ALT/AST, 9 (11.1%) missing platelet count, 2 (2.5%) had missing glucose/cholesterol, one (1.1%) had missing glucose, and another one (1.1%) missing GGT.

To compare disease severity, we compared median LSM between included and excluded patients in both cohorts. In cohort I, the median LSM was 6.9 [IQR: 5.1-10.9]kPa for included and 5.9 [IQR: 4.5-7.9]kPa for excluded patients showing a minor but statistically significant difference in terms of a lower LSM in patients with missing laboratory values (Mann-Whitney U test $p<0.001$). In cohort II, no difference in median LSM was observed (5.8 [IQR: 4.5-8.8]kPa in included vs. 6.5 [IQR: 5.0-10.3]kPa in excluded patients [$p=0.090$]).

Supplementary figures

Fig. S1. Scatterplot and LOESS curves of LiverRisk score and LSM, FIB-4, and APRI in cohort I (**A**) and cohort II (**B**).

A

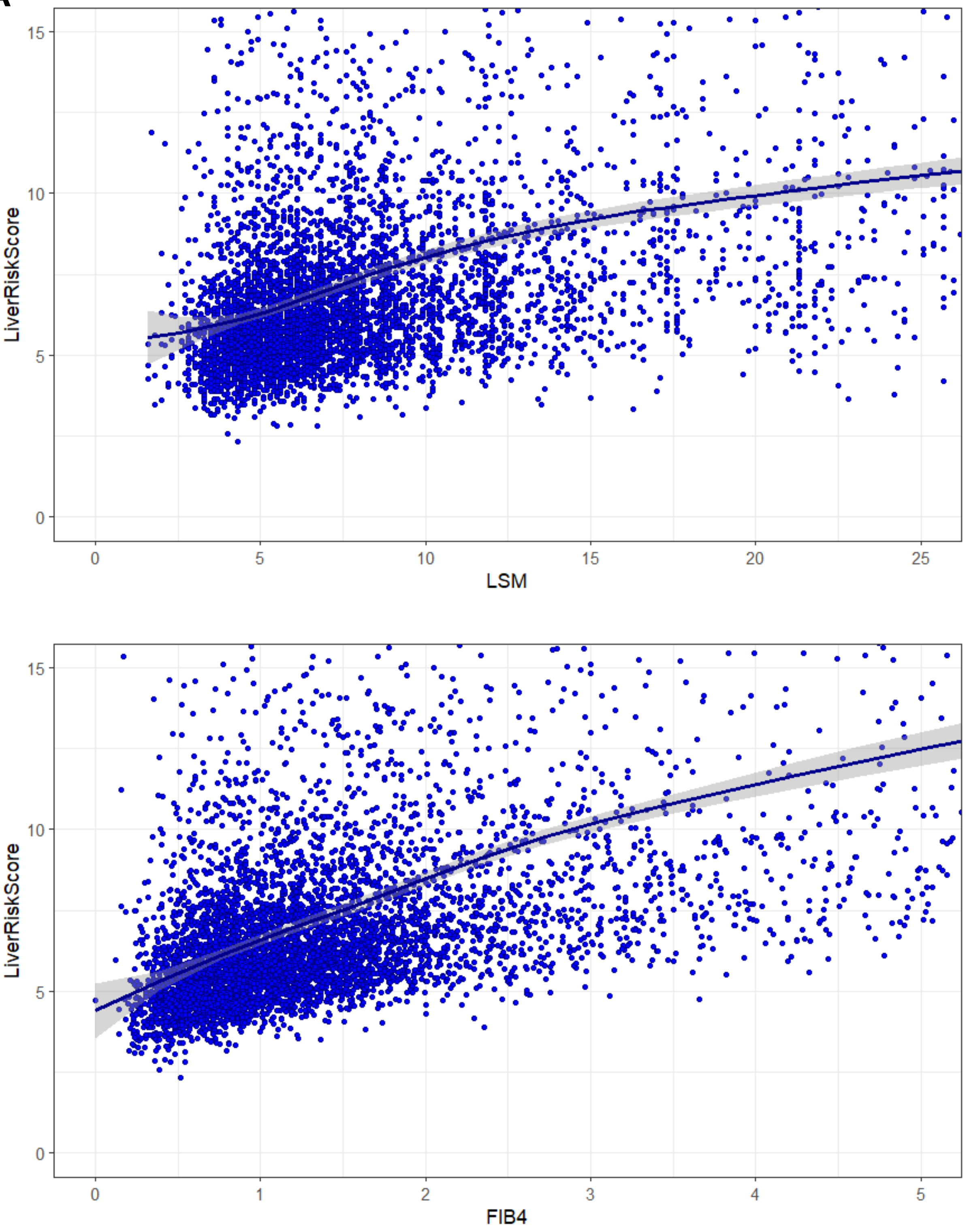

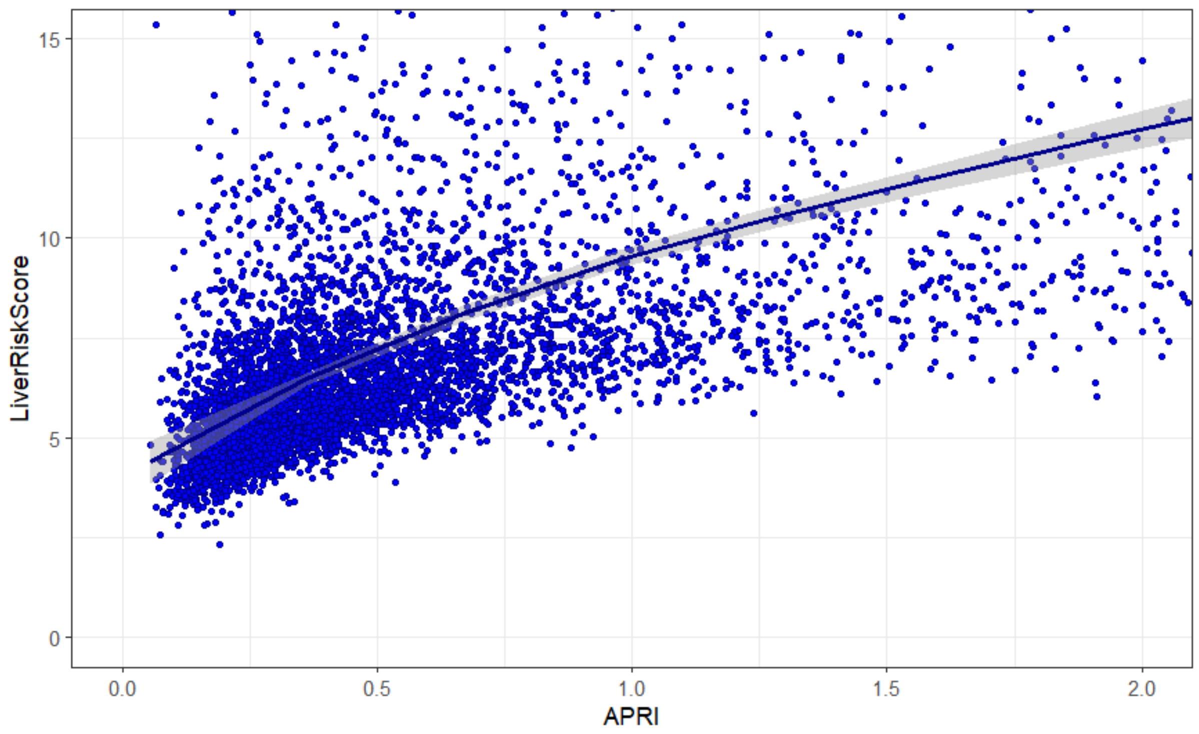
15
10
5
0
0.0
0.5
1.0
1.5
2.0
LiverRiskScore
APRI

B

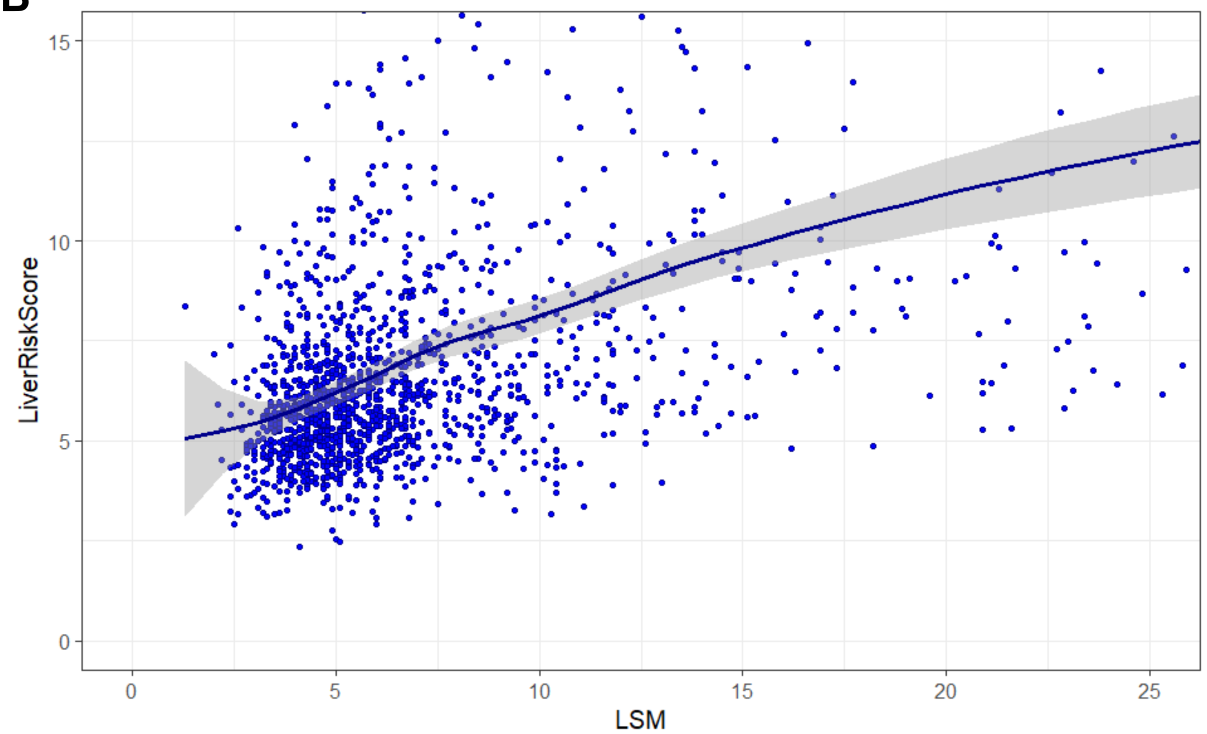
15
10
5
0
0
5
10
15
20
25
LiverRiskScore
LSM

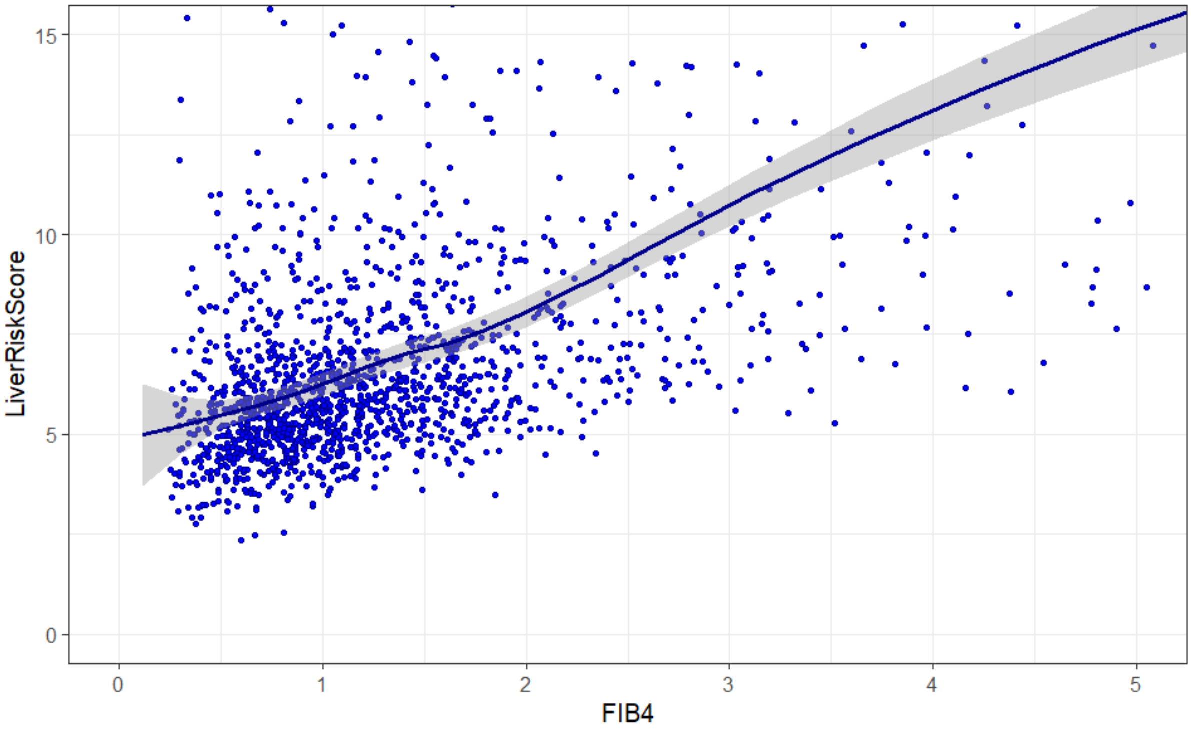

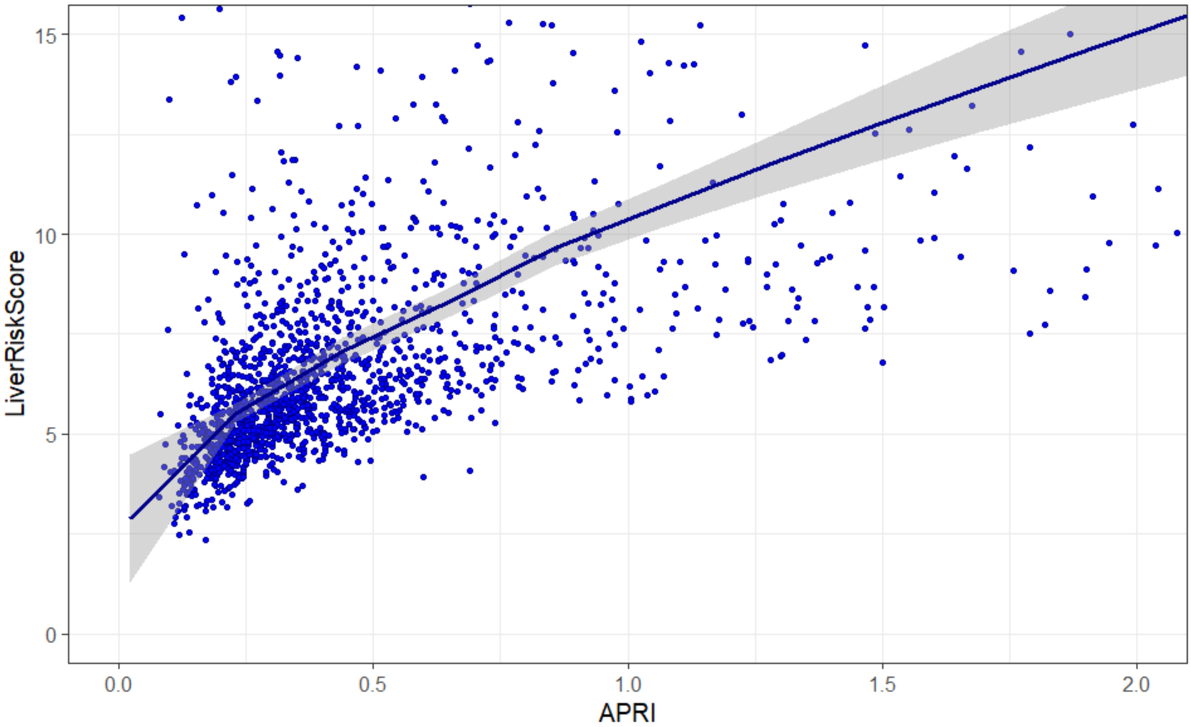

Abbreviations: APRI – aspartate aminotransferase to platelet ratio index; cACLD – compensated advanced chronic liver disease; FIB-4 – fibrosis 4 score; LOESS - locally estimated scatterplot smoothing; LSM – liver stiffness measurement;

Fig. S2. Receiver operating characteristics (ROC) curves for the detection of cACLD (LSM ≥10kPa) using LiverRisk score, FIB-4, and APRI in cohort I (**A**) and cohort II (**B**).

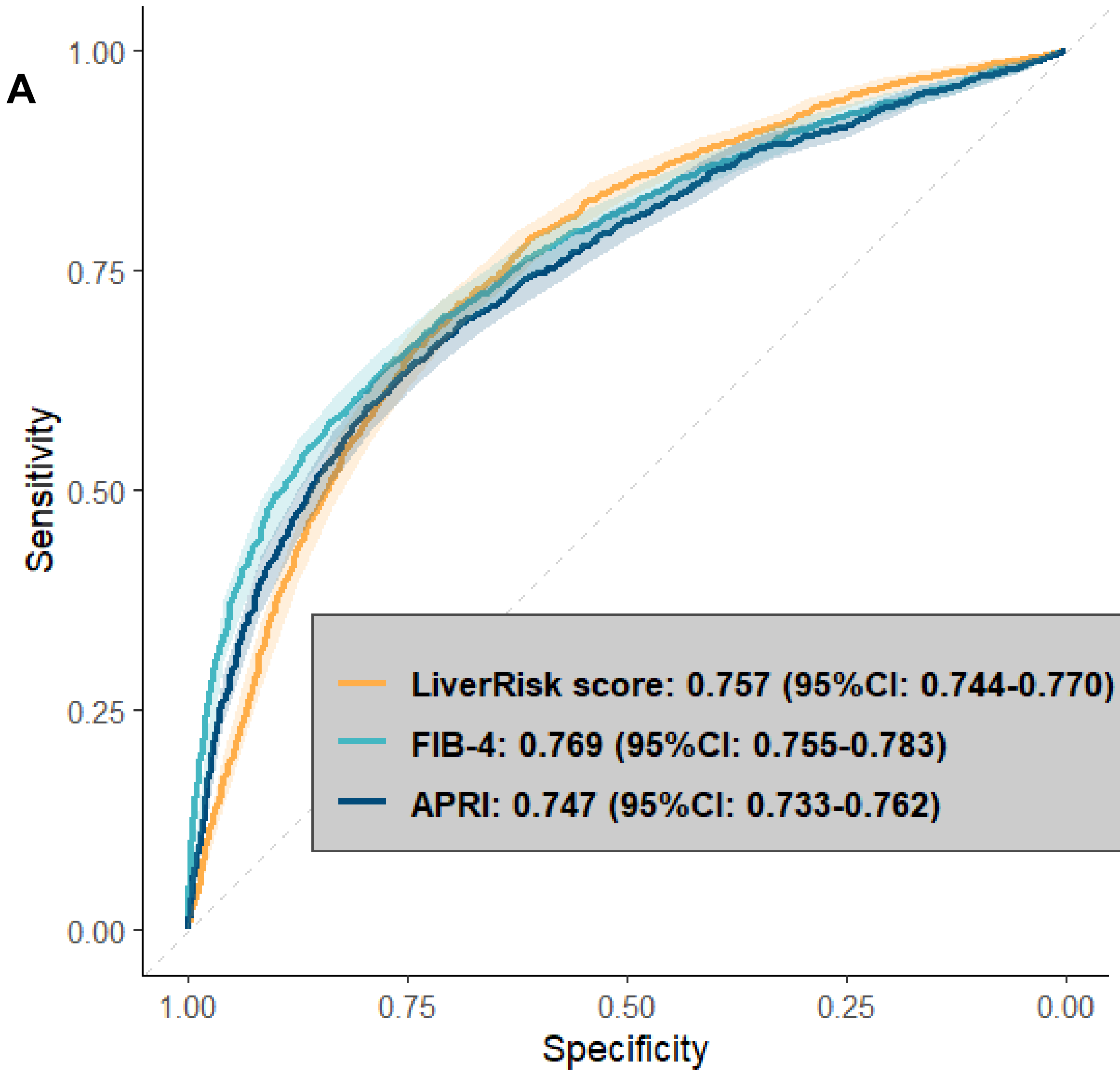

B

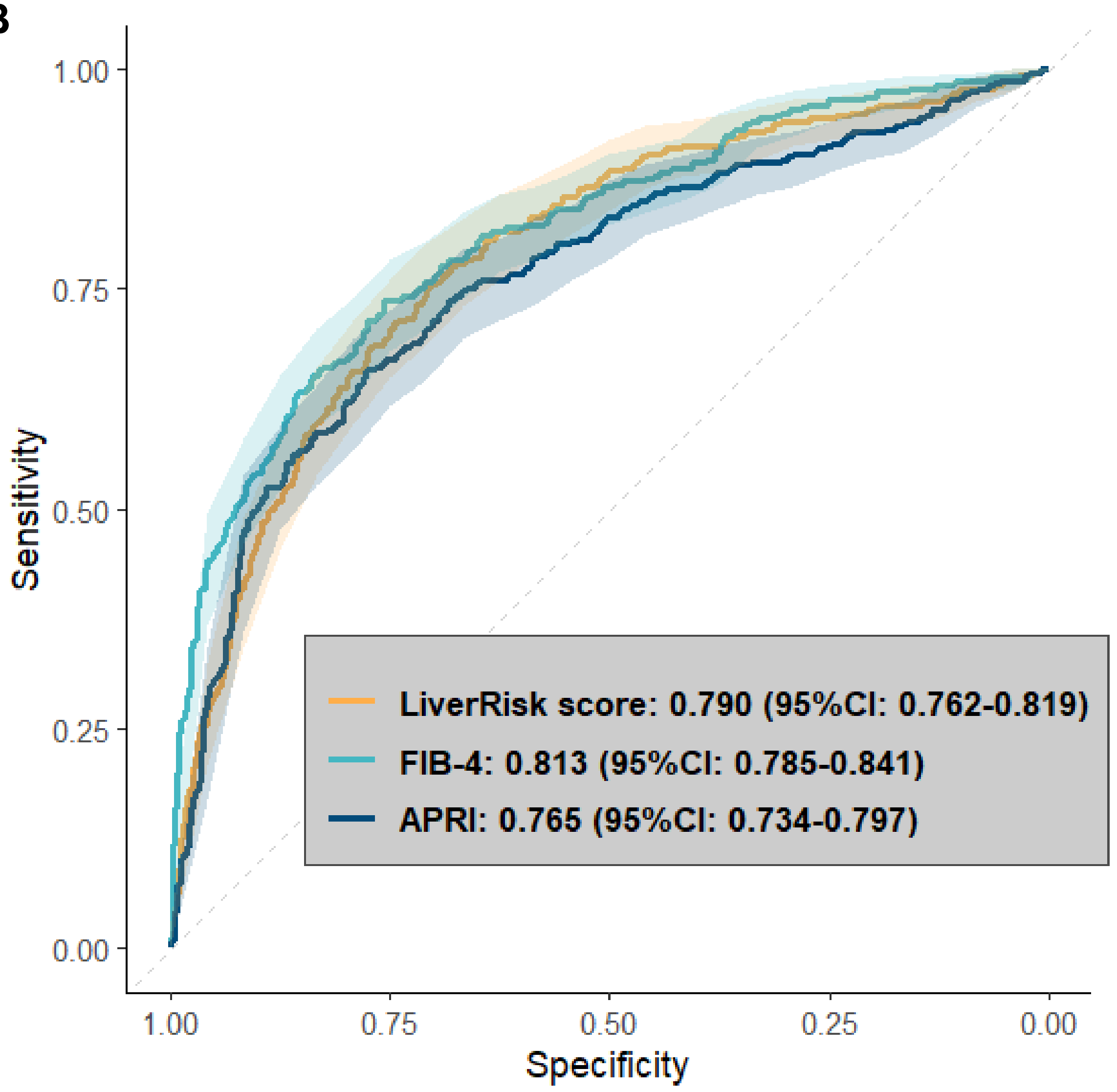

Abbreviations: APRI – aspartate aminotransferase to platelet ratio index; cACLD – compensated advanced chronic liver disease; FIB-4 – fibrosis 4 score; LSM – liver stiffness measurement; ROC – receiver operating characteristics;

Fig. S3. Time-dependent area under the receiver operator characteristics curves (AUROC) and 95% confidence intervals (95%CI) of LiverRisk score, LSM, FIB-4, and APRI for the prediction of hepatic decompensation in cohort I at 1-5 years of follow-up.

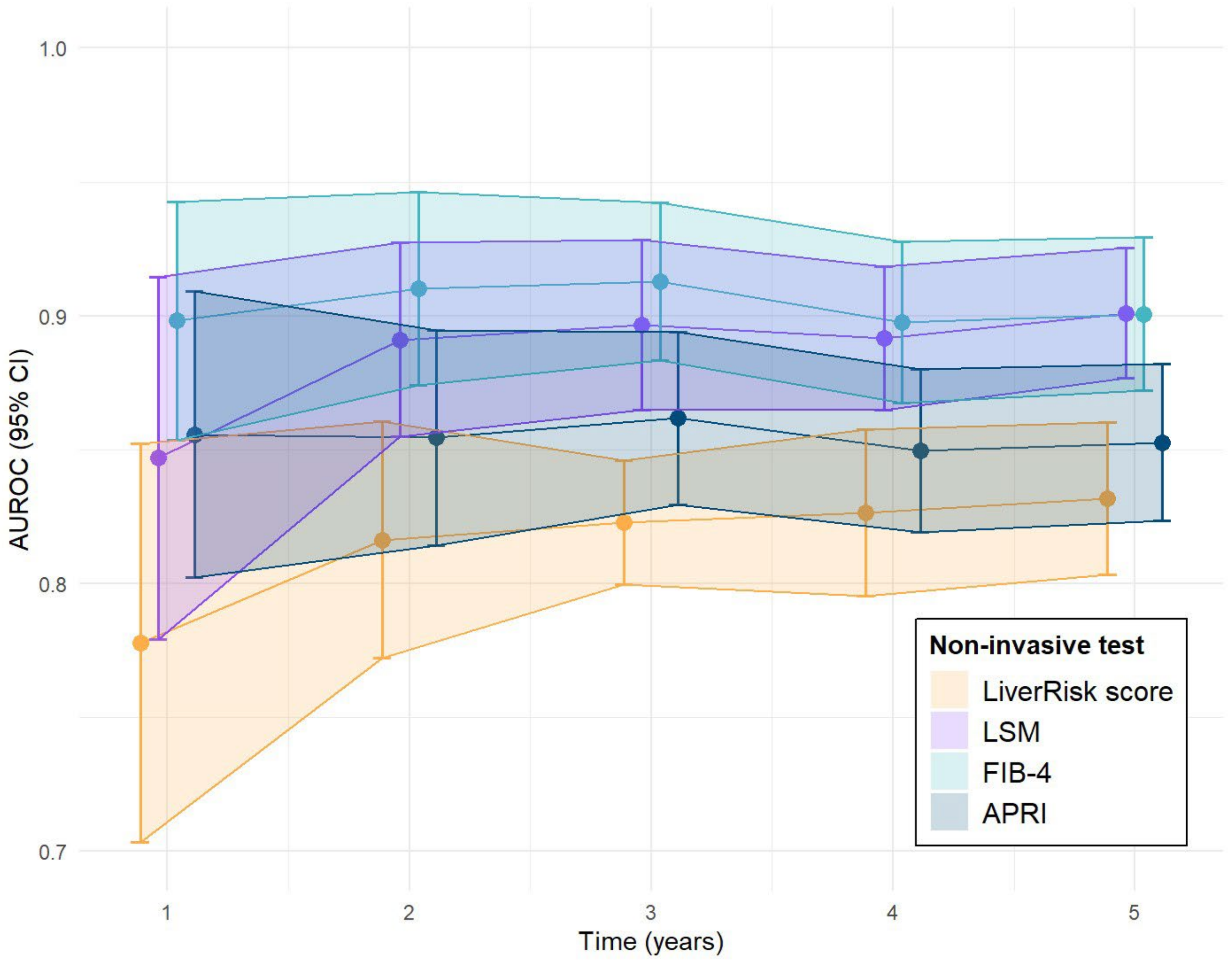

Abbreviations: AUROC – area under the receiver operator characteristics curve; APRI – aspartate aminotransferase to platelet ratio index; cACLD – compensated advanced chronic liver disease; FIB-4 – fibrosis 4 score; LSM – liver stiffness measurement; 95%CI – 95% confidence interval;

Fig. S4. Cumulative incidence curves of hepatic decompensation compared across categories of LiverRisk score (<6, 6-<10, 10-<15, ≥15) in cohort I. Subdistribution hazard ratios (SHR) are given as compared to the first group. (Fine-Gray subdistribution hazard model)

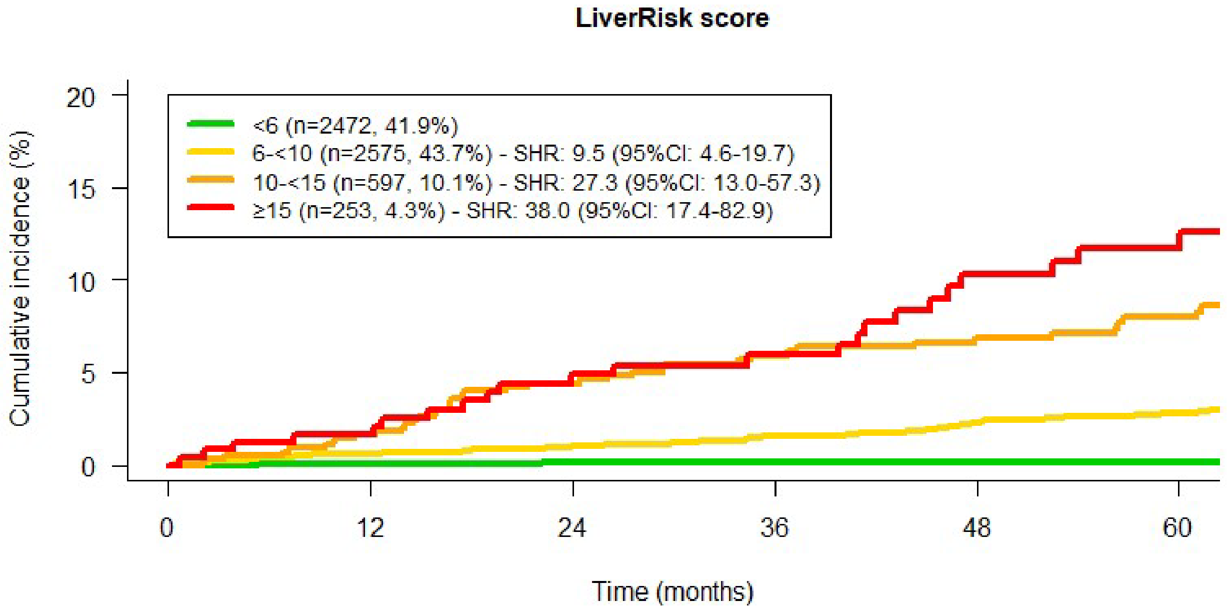

Abbreviations: LSM – liver stiffness measurement; SHR – subdistribution hazard ratio;

Supplementary tables

Table S1. Correlation matrix (Spearman's rank correlation coefficient ρ) for LiverRisk score, LSM, FIB-4, and APRI in cohort I and cohort II.

**Cohort I**	**LiverRisk score**	**LSM**	**FIB-4**	**APRI**
LiverRisk score	1.000	0.469	0.577	0.711
LSM	0.469	1.000	0.448	0.452
FIB-4	0.577	0.448	1.000	0.722
APRI	0.711	0.452	0.722	1.000
**Cohort II**	**LiverRisk score**	**LSM**	**FIB-4**	**APRI**
LiverRisk score	1.000	0.471	0.535	0.684
LSM	0.471	1.000	0.419	0.415
FIB-4	0.535	0.419	1.000	0.617
APRI	0.684	0.415	0.617	1.000

Abbreviations: APRI – aspartate aminotransferase to platelet ratio index; FIB-4 – fibrosis 4 score; LSM – liver stiffness measurement;

Table S2. Cohen's Kappa and 95% confidence intervals (95%CI) between LSM ≥10kPa and LiverRisk score ≥10points in cohort I and cohort II both in the overall cohorts as well as across etiology subgroups.

	**Cohort I, n=5897**	**Cohort II, n=1558**
Overall cohort	0.260 (0.233-0.286)	0.327 (0.268-0.385)
MASLD	0.237 (0.183-0.292)	0.338 (0.257-0.420)
ALD	0.210 (0.110-0.309)	0.337 (0.190-0.483)
Viral	0.255 (0.220-0.290)	0.143 (0.023-0.263)
AIH/Cholestatic	0.309 (0.217-0.401)	0.330 (0.122-0.538)
Other	0.150 (0.030-0.270)	-0.071 (-0.345-0.204)

Abbreviations: AIH – autoimmune hepatitis; ALD – alcohol-related liver disease; LSM – liver stiffness measurement; MASLD – metabolic dysfunction-associated steatotic liver disease; 95%CI – 95% confidence interval;

Table S3. Correlation, calibration, and agreement metrics between the LiverRisk score and LSM, as well as metrics on the diagnostic accuracy of LiverRisk score for the diagnosis of cACLD in cohort I and cohort II in the subgroup of patients meeting reliability criteria for liver fibrosis assessment (≤7kPa or IQR/median <0.3). (Pearson's r, linear regression, Bland-Altmann analysis, ROC analysis)

Out-come	Metric	Cohort I, n=5509	Cohort II, n=1430
LSM (kPa, linear)	Pearson's r (correlation, strong calibration)	0.323	0.431
	R^2 (variation, strong calibration)	0.104	0.185
	Intercept (weak calibration)	4.225 (SE: 0.265)	1.354 (SE: 0.451)
	Slope (weak calibration)	0.775 (SE: 0.031)	0.977 (SE: 0.054)
	Mean difference (95%CI)[1] ≙ Calibration in the large (mean calibration)	2.54 (2.28-2.80)	1.19 (0.75-1.63)
	Lower limit of agreement (95%CI)[1]	-16.87 (-17.32-[-16.42])	-15.57 (-16.33-[-14.82])
	Upper limit of agreement (95%CI)[1]	21.95 (21.50-22.40)	17.96 (17.20-18.71)
	Interval of agreement (kPa/points; 95%CI)[1]	38.82	33.53
cACLD (≥10kPa)	Prevalence (%)	1423 (25.8%)	242 (16.9%)
	AUROC (95%CI)	0.765 (0.751-0.779)	0.816 (0.786-0.845)
	Sensitivity[2] (95%CI)	31.3% (28.9-33.8%)	38.0% (31.9-44.5%)
	Specificity[2] (95%CI)	92.0% (91.1-92.8%)	93.2% (91.6-94.6%)
	Positive predictive value[2] (95%CI)	57.6% (54.4-60.7%)	53.2% (46.6-59.7%)
	Negative predictive value[2] (95%CI)	79.4% (78.8-80.0%)	88.1% (87.0-89.1%)
	Accuracy[2] (95%CI)	76.3% (75.2-77.4%)	83.9% (81.8-85.7%)

[1] based on Bland-Altmann analysis (LSM – LiverRisk score); [2] applying at cut-off of 10points corresponding to 10kPa

Abbreviations: AUROC – area under the receiver operator characteristics curve; cACLD – compensated advanced chronic liver disease; LSM – liver stiffness measurement; ROC – receiver operator characteristics; SE – standard error; 95%CI – 95% confidence interval;

Table S4. Area under the receiver operator characteristics curve (AUROC) and 95% confidence intervals (95%CI) of LiverRisk score, FIB-4 and APRI for the diagnosis of cACLD in cohort I and cohort II, as well as time-dependent AUROC of LiverRisk score, LSM, FIB-4 and APRI for the prediction of hepatic decompensation in cohort I in the subgroup of patients meeting reliability criteria for liver fibrosis assessment (≤7kPa or IQR/median <0.3). (ROC analysis, time-dependent ROC analysis, comparison according to Blanche et al [2013], Level of significance after multiplicity correction: p<0.05)

Outcome	Metric	Cohort I, n=5509	Cohort II, n=1430
cACLD (≥10kPa)	LiverRisk score	0.765 (0.751-0.779)	0.816 (0.786-0.845)
	FIB-4	0.782 (0.767-0.797)	0.826 (0.796-0.857)
	APRI	0.760 (0.745-0.775)	0.781 (0.746-0.816)

Outcome	Metric	Cohort I, n=5509				
Hepatic decompensation	Time	1 year	2 years	3 years	4 years	5 years
	Number of events	24	51	70	92	101
	LiverRisk score	0.772 (0.690-0.855)	0.809 (0.760-0.858)	0.820 (0.782-0.858)	0.829 (0.797-0.862)	0.833 (0.802-0.863)
	LSM	0.860 (0.786-0.934)*	0.898 (0.859-0.937)*	0.904 (0.872-0.937)*	0.901 (0.875-0.928)*	0.906 (0.882-0.931)*
	FIB-4	0.905 (0.860-0.951)*	0.909 (0.869-0.949)*	0.913 (0.881-0.945)*	0.896 (0.864-0.928)*	0.903 (0.874-0.932)*
	APRI	0.862 (0.804-0.919)	0.855 (0.810-0.900)	0.864 (0.829-0.899)	0.851 (0.819-0.882)	0.852 (0.820-0.884)

[*] Indicates a statistically significant difference in AUROC as compared to the LiverRisk score (according to Blanche et al [2013])

Abbreviations: AUROC – area under the receiver operator characteristics curve; cACLD – compensated advanced chronic liver disease; LSM – liver stiffness measurement; ROC – receiver operator characteristics; 95%CI – 95% confidence interval;
